# Evaluation of Prenatal Transportation Stress on DNA Methylation (DNAm) and Gene Expression in the Hypothalamic–Pituitary–Adrenal (HPA) Axis Tissues of Mature Brahman Cows

**DOI:** 10.3390/genes16020191

**Published:** 2025-02-04

**Authors:** Audrey L. Earnhardt-San, Emilie C. Baker, Kubra Z. Cilkiz, Rodolfo C. Cardoso, Noushin Ghaffari, Charles R. Long, Penny K. Riggs, Ronald D. Randel, David G. Riley, Thomas H. Welsh

**Affiliations:** 1 Department of Animal Science, Texas A&M University, College Station, TX 77843, USA; audrey.san@delval.edu (A.L.E.-S.); ebaker@wtamu.edu (E.C.B.); kubrazozik@tamu.edu (K.Z.C.); r.cardoso@tamu.edu (R.C.C.); charles.long@ag.tamu.edu (C.R.L.); riggs@tamu.edu (P.K.R.); ron.randel@ag.tamu.edu (R.D.R.); david.riley@ag.tamu.edu (D.G.R.); 2 Texas A&M AgriLife Research Center, Overton, TX 75684, USA; 3 Department of Computer Science, Prairie View A&M University, Prairie View, TX 77070, USA; noghaffari@pvamu.edu

**Keywords:** prenatal stress, HPA axis, DNA methylation, gene expression, cattle

## Abstract

**Background/Objectives:** The experience of prenatal stress results in various physiological disorders due to an alteration of an offspring’s methylome and transcriptome. The objective of this study was to determine whether PNS affects DNA methylation (DNAm) and gene expression in the stress axis tissues of mature Brahman cows. **Methods:** Samples were collected from the paraventricular nucleus (PVN), anterior pituitary (PIT), and adrenal cortex (AC) of 5-year-old Brahman cows that were prenatally exposed to either transportation stress (PNS, *n* = 6) or were not transported (Control, *n* = 8). The isolated DNA and RNA samples were, respectively, used for methylation and RNA-Seq analyses. A gene ontology and KEGG pathway enrichment analysis of each data set within each sample tissue was conducted with the DAVID Functional Annotation Tool. **Results:** The DNAm analysis revealed 3, 64, and 99 hypomethylated and 2, 93, and 90 hypermethylated CpG sites (FDR < 0.15) within the PVN, PIT, and AC, respectively. The RNA-Seq analysis revealed 6, 25, and 5 differentially expressed genes (FDR < 0.15) in the PVN, PIT, and AC, respectively, that were up-regulated in the PNS group relative to the Control group, as well as 24 genes in the PIT that were down-regulated. Based on the enrichment analysis, several developmental and cellular processes, such as maintenance of the actin cytoskeleton, cell motility, signal transduction, neurodevelopment, and synaptic function, were potentially modulated. **Conclusions:** The methylome and transcriptome were altered in the stress axis tissues of mature cows that had been exposed to prenatal transportation stress. These findings are relevant to understanding how prenatal experiences may affect postnatal neurological functions.

## 1. Introduction

Stress is the combination of the biochemical, physiological, and behavioral responses by which animals and humans cope with real or perceived threats to homeostasis. When animals are exposed to a stressful stimulus, the response that is triggered is multifaceted, in that it involves neural, neuroendocrine, and endocrine systems to deal with the stressor and return the body to normal [1,2]. Specific pathways are activated to mediate this response, which occur through the sympathomedullary system (SMS) and the hypothalamic–pituitary–adrenal (HPA) axis, resulting in increased catecholamine and glucocorticoid secretion [3,4,5]. Elevated maternal glucocorticoid, or cortisol, is thought to potentially affect fetal programming through either direct or indirect means, resulting in the frequent observation of stress axis dysregulation and an increased risk of morbidity in offspring [6,7]. Though the mechanisms behind this programming are still unclear, several studies have suggested that increased maternal cortisol secretion, particularly during chronic stress, may impair the metabolism of cortisol within the placenta by the barrier enzyme 11β-hydroxysteroid dehydrogenase type 2 [8,9,10]. This impairment exposes the fetus to greater concentrations of cortisol, altering the fetal environment, and through either direct or indirect means potentially affects the fetal response to a specific insult during a critical period of prenatal development [6,7].

Maximal epigenetic plasticity occurs during the prenatal period through weaning in mammals [11,12], where the epigenetic mechanisms mediate development through the regulation of gene expression [13]. With a potential impaired placental maintenance of the fetal environment during maternal stress, a fetus is vulnerable to epigenetic modifications, such as DNA methylation (DNAm), during critical points of development [14]. DNAm plays a major role in regulating distinct functions among different cell types, despite the shared identical genome [15]. Various animal and human studies have shown that the regulation of gene expression through DNA methylation is key to connecting prenatal stress to inapt outcomes of the offspring. A comparison of cattle with divergent temperament (i.e., docile versus excitable) identified the following: (1) the differential methylation of DNA in specific regions of the brain [16], (2) differential gene expression in the adrenal cortex [17], and (3) differences in the abundance of metabolites in the blood serum and the brain prefrontal cortex [18]. Takahashi et al. [19] showed that pregnant rats stressed by electric tail shock gave birth to offspring with increased plasma corticosterone, while Littlejohn et al. [20] found that Brahman calves, a tropically adapted breed with tolerance to elevated heat and humidity, exposed to prenatal transportation stress were more temperamental and had elevated serum concentrations of cortisol relative to the controls. Lymphocyte DNAm profiles were altered as early as 28 days of age in calves exposed to prenatal transportation stress [21,22], and differentially methylated stress-response genes were seen in prenatally stressed bull calves [23]. A genome-wide DNAm analysis of the lymphocytes of prenatally stressed Brahman cows compared to controls also revealed an altered epigenome at 5 years of age [24]. Although several studies have focused on the relationship between stress and DNAm and gene expression within the peripheral blood lymphocytes, due to restricted accessibility there are limited studies on the effect of stress on DNAm and gene expression in the HPA axis tissues.

Gestating cows may be exposed to various stressors in a typical cow–calf production setting, such as handling, restraint, or transportation [7,25]. Transportation has been known and confirmed [26,27] to be a stressor for pregnant Brahman cows, resulting in elevated body temperature and systemic concentrations of cortisol and glucose. The findings of Price et al. [27] reaffirmed reports that transportation constitutes a stressor for pregnant cattle and could thereby influence postnatal development and physiology [28,29]. We hypothesized that there would be differences in DNAm and gene expression within the primary tissues of the stress axis between the prenatally transported and Control cows at 5 years of age, similar to the altered DNA methylation profiles and gene expression seen in the lymphocytes of Brahman heifer calves with the advancement of age [24]. Specifically, we compared DNAm and gene expression within the key stress axis tissues isolated from (1) the cows that experienced prenatal transportation stress (PNS) in utero and (2) their Control cohorts. This study was undertaken because changes in methylation patterns or gene expression due to prenatal stress could alter biological pathways in ways that negatively impact postnatal cattle health, behavior, and production traits important to producers and consumers, resulting in economic and welfare problems.

## 2. Materials and Methods

### 2.1. Animal Procedures

All procedures complied with the Guide for the Care and Use of Agricultural Animals in Research and Teaching [30] and were approved by the Texas A&M AgriLife Research Animal Use and Care Committee (Animal Use Protocol # 2017-001A). As described previously [21,24,27], artificially inseminated and confirmed-pregnant Brahman cows (*n* = 48) were transported in a three-section trailer on smooth highways for a 2-h period (at an average speed of 75 km/h) at 60 ± 5, 80 ± 5, 100 ± 5, 120 ± 5, and 140 ± 5 days of gestation. Low-stress handling techniques were followed to load cows on and off the trailer. In our PNS model, day 60 of gestation is the earliest time point to reduce the risk of losing the pregnancy [31]. Day 140 is the latest time point to avoid premature labor due to stress-induced elevation in secretion of fetal adrenal cortisol [32,33]. Pregnant cows that were not transported (*n* = 48) were designated as the Control group. Both the transported and Control cows were kept in the same pasture under the same environmental and nutritional conditions at the Texas A&M AgriLife Research & Extension Center in Overton, TX (32.27° N, −94.98° W). The transported cows gave birth to 20 male and 21 female calves (prenatal transportation stress group, PNS), while the Control cows gave birth to 26 male and 18 female calves (Control). The 18 Control and 21 PNS female calves were kept in the same pasture and nutritional conditions at the Texas A&M AgriLife Research & Extension Center in Overton until they averaged 5 years of age, when 8 nonpregnant cows from each group were selected at random for tissue collection. Eight cows per group were selected because previous publications had detected differences in both bovine leukocyte and brain cell DNA methylation patterns with five to seven individuals per treatment group [16,21,22]. The 5-year-old nonpregnant PNS (*n* = 6) and Control (*n* = 8) Brahman cows were trailered to the departmental cattle facility and held for approximately 15 h in pens with free access to water before processing at the departmental abattoir. In the presence of a trained inspector from the Texas Department of Agriculture, the cows were harvested humanely between 0800 and 1100 h to obtain tissues of the stress axis. The abattoir staff members, trained in low-stress handling methods, quietly eased the cows into the stun box. The staff promptly stunned the cows by using a captive bolt. By use of a band saw the brain was removed rapidly to collect the hypothalamus and pituitary gland. The adrenal glands were collected within 15 min of exsanguination. The cows were processed in a balanced alternating order (Control, PNS, Control, PNS, etc.) to minimize the potential confounders of time and order of processing. Animal identification and treatment group designation were known to the investigators since the animals were born. However, tissues were collected from fewer PNS than Control cows because two pregnant PNS cows were mistakenly transported to the cattle facility rather than the intended two nonpregnant PNS cows. This error was discovered when animal identification was confirmed before transport to the abattoir. The sample tissues, including the paraventricular nucleus (PVN), anterior pituitary gland (PIT), and adrenal cortex (AC), were promptly isolated, processed on site, stored in RNase/DNase-free sterile cryovials, snap-frozen in a portable liquid nitrogen tank, and then moved to the research laboratory in an adjacent building for storage at −80 °C.

### 2.2. Collection of Sample Tissues from the Stress Axis

To collect tissue containing the paraventricular nucleus (PVN), a block of tissue containing the septum, preoptic area, and hypothalamus of the brain was dissected, immediately frozen in dry ice, and then stored at −80 °C until further processing. The frozen blocks of hypothalamic tissue were cut into coronal sections of 20 μm using a Leica CM1900 cryostat (Leica Biosystems, Nufloch, Germany), and tissue sections were thaw-mounted on Superfrost/Plus glass microscope slides (Fisher Scientific, Waltham, MA, USA), frozen immediately, and stored at −80 °C until further processing. To determine the location of the PVN, a single series of hypothalamic tissue sections containing sections 200 μm apart was processed for Cresyl violet staining and observed using bright-field microscopy. The location was determined through the identification of well-established anatomical landmarks, including the third ventricle and the anterior commissure [34,35].

For tissue dissection, as well as DNA and RNA isolation, a separate series of sections containing the PVN was used. An area of approximately 1 mm in diameter encompassing the PVN was scraped while frozen from the slides using a 25-gauge needle (12 sections from each animal were used for extraction of at least 1–4 μg total RNA for sequencing procedures, and 25–30 sections from each animal were used for extraction of at least 25 ng/μL DNA for methylation analysis procedures). Tissue for DNA analysis immediately went through DNA isolation protocols and tissue for RNA analysis was stored at −80 °C.

### 2.3. DNA and RNA Extraction

The PVN tissues were digested in a solution containing 250 μL of STE buffer, 25 μL of Proteinase K (20 mg/mL), and 25 μL 20% SDS. Sample tubes were incubated in a water bath at 56 °C for 2 h, then 20 μL of RNAse A (10 mg/mL) was added to sample tubes and mixture was incubated at 37 °C for 30 min. DNA from digested PVN tissues was isolated using a modified version of the phenol/chloroform/isoamyl alcohol DNA purification protocol described by Strauss [36]. DNA from PIT and AC tissues was isolated using the manufacturer’s protocol for a GeneJET Genomic DNA Purification Kit (Thermo Scientific, Waltham, MA, USA). Prior to DNA isolation, tissues were digested in a water bath at 56 °C, with PIT tissue being digested for 6 h, AC tissue for 2.75 to 3.5 h, and AM tissue for 2.5 to 3.5 h. Purified DNA samples were quantified with a NanoDrop Spectrophotometer (NanoDrop Technologies, Rockland, DE, USA) and stored at −80 °C until further analysis.

Frozen tissue was submitted to Texas A&M Institute for Genome Sciences and Society (TIGSS) for isolation of total RNA with a TRIzol Plus RNA Purification Kit (Thermo Scientific, Waltham, MA, USA). An Illumina TruSeq Stranded Total RNA kit was used to process the PIT and AC RNA. The purified RNA was quantified with a Qubit RNA Fluorometric Assay Kit (Thermo Scientific, Waltham, MA, USA) and the RNA quality was assessed with an Agilent 4200 Tape Station System (Software 5.1; Agilent Technologies, Santa Clara, CA, USA). An Agilent RNA ScreenTape^®^ assay for eukaryotic RNA with a nucleotide reference ladder (25, 200, 500, 1000, 2000, 4000, and 6000 nt) was used to obtain the RNA integrity number (RINe).

### 2.4. DNA Methylation Library Construction and Alignment

Purified DNA samples were submitted to the Zymo Research Corporation (Irvine, CA, USA). The submitted samples were coded so that the technicians were blinded regarding treatment group until data were analyzed. DNA samples were processed and analyzed using the Methyl-MiniSeq Service: Genome-wide bisulfite sequence. From the generated Methyl-MiniSeq libraries, sequence reads were identified using standard Illumina base-calling software (Illumina Inc., San Diego, CA, USA). Raw FASTQ files were adapted and quality-trimmed using TrimGalore 0.6.4, which was also used to trim filled-in nucleotides. Overall quality distribution of the data and the effect of trimming were assessed using FastQC 0.11.8. Alignment with the ARS-OCD1.2/bosTau9 reference genome was performed using Bismarck 0.19.0. The methylated and unmethylated read totals for each CpG (5’-C-phosphate-G-3’) site were called using MethylDackel 0.5.0.

### 2.5. RNA Sequencing and Annotation

The samples submitted to TIGSS were coded so that the technicians were blinded regarding treatment group until data were analyzed. The TIGSS personnel processed the PVN and PIT total RNA samples using the HS protocol of an Illumina TruSeq Stranded mRNA library preparation kit with a Dual Indexed RNA Adapter Plate (Illumina Inc., San Diego, CA, USA). For the AC total RNA samples, TIGSS utilized globin and ribosomal RNA depletion polyA selection for mRNA isolation with the TruSeq Stranded Total RNA Library Prep workflow with Ribo-Zero Globin (Illumina Inc., San Diego, CA, USA). Libraries were validated with an Agilent 4200 TapeStation which determined the average base pair size of the sample. Cluster generation and sequencing were performed on a NovaSeq 6000 Sequencing System in paired-end, 150 bp cycles (Illumina Inc., San Diego, CA, USA).

### 2.6. Preparation of Raw Data for Analysis

Analysis performed by Zymo Research Corp. produced methylation call tables that consisted of the chromosome and base pair position of each CpG site analyzed, as well as the methylation coverage and total coverage at those sites. For the files to be in the proper format for edgeR methylation analysis, adjustments were made to the methylation call tables in Terminal on the macOS Big Sur 11.1 operating system. Percent methylation, methylated counts, and unmethylated counts were calculated for each site utilizing the methylation coverage count and the total coverage count. Percentage methylation was calculated by dividing the methylation coverage count by the total coverage count. Methylated count was simply the methylation coverage count, and unmethylated count was the total coverage count minus the methylated coverage count.

Raw gene counts were generated from the resulting RNA-Seq FastQ files by trimming the read adapters with Trimmomatic 0.38 [37]. The trimmed reads were then mapped to a *Bos taurus* reference genome (Umd3.1), and the resulting mapped reads were counted using featureCounts 1.22.2 [38]. After generation of raw gene counts, the counts for each individual animal were combined into a single comma-separated file for each tissue, with the Ensembl gene ID in the first column. These files were then used for further analysis with the edgeR Package (v. 3.13) from Bioconductor in R [39]. The raw gene count tables were read into rStudio (v. 4.2.2), and the low-expression genes were filtered out using the criteria of 3 minimum reads per gene in at least 5 samples. The normalization factors were then calculated using the trimmed mean of M-values (TMM) method to produce normalized gene counts within each sample tissue.

### 2.7. Differential DNA Methylation and Gene Expression Analysis and Annotation

Using the reformatted methylation call tables, the methylation analysis was performed in RStudio using the edgeR Package. The sites were filtered so that only the sites that had 8× coverage in every sample were analyzed. A negative binomial generalized log-linear model was fitted to the read counts of the data set, and site-wise statistical tests were conducted for the contrast of PNS group minus Control group, where CpG sites were hypermethylated (hypomethylated) if PNS group had more (less) methylation than Control group. The Benjamin–Hochberg method for multiple test correction was applied to control false discoveries. Significant sites were further analyzed utilizing the Ensembl BioMart tool (Ensembl Release 108).

Analysis of differentially expressed genes between Control and PNS animals in each tissue was performed with the edgeR Package in RStudio (v. 4.2.2). The raw gene count tables were read into RStudio and animals were grouped based on treatment (Control versus PNS). Low-expressed genes were filtered out using the criteria of 3 minimum reads per gene in at least 5 samples. Normalization factors were then calculated using the trimmed mean of M-values (TMM) method. The design matrix of the statistical test was defined for animals and their treatment group, and dispersion was estimated with the generalized linear model (GLM) method. A negative binomial model was fitted to the dispersion estimates to use the GLM likelihood ratio test using the contrast of PNS group minus Control group, where genes were up-regulated (down-regulated) if PNS group had increased (decreased) gene expression relative to Control group. The topTags function was utilized to extract genes that were differentially expressed from the data results.

For both DNA methylation and gene expression data, changes of at least two-fold with an FDR < 0.05 were considered significant. Due to limited results for multiple tissues, the stringency for differential methylation and differential expression was reduced by allowing an FDR < 0.15 (Table 1 and Table 2). Gene ontology (GO) and pathway enrichment analyses of resulting differentially methylated genes (DMGs) and differentially expressed genes (DEGs) were performed using DAVID Bioinformatic Resources (https://david-d.ncifcrf.gov/home.jsp (accessed on 18 November 2024)) in order to determine common results across platforms [40,41]. Enrichment results were visualized using the ggplot2 Package in RStudio (v. 4.2.2).

## 3. Results

### 3.1. Identification of DMGs and DEGs

**Figure 1:**
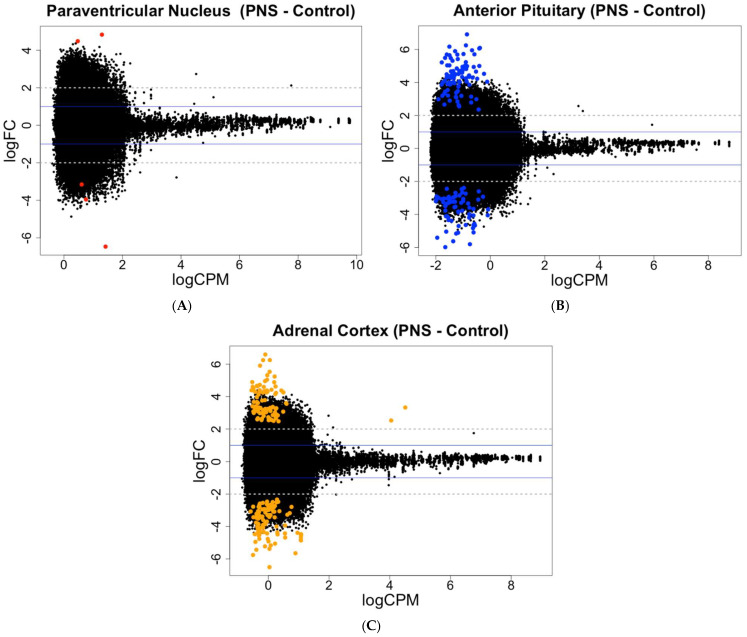
MA plots showing the relationship between the average concentration (logCPM) and fold-change (logFC) across the CpG sites in each tissue. Each site is represented by a dot with differentially methylated CpG sites (FDR < 0.15) colored in (**A**) red for the PVN, (**B**) blue for the PIT, and (**C**) orange for the AC. The blue solid lines represent a logFC ± 1 threshold, and the gray dotted lines represent a logFC ± 2 threshold.

In the PNS group, relative to the Control group, the number of differentially methylated CpG sites within the PVN did not change with reduced stringency, while the PIT differentially methylated CpG sites more than doubled from 73 to 157 (FDR < 0.05 and FDR < 0.15, respectively). In the AC, the number of differentially methylated CpG sites increased from 103 to 189 with reduced stringency (FDR < 0.05 and FDR < 0.15, respectively). In contrast to the PVN and AC, the PIT had greater amounts of hypermethylated than hypomethylated sites (FDR < 0.15; Figure 1; Supplementary Table S1).

**Figure 2:**
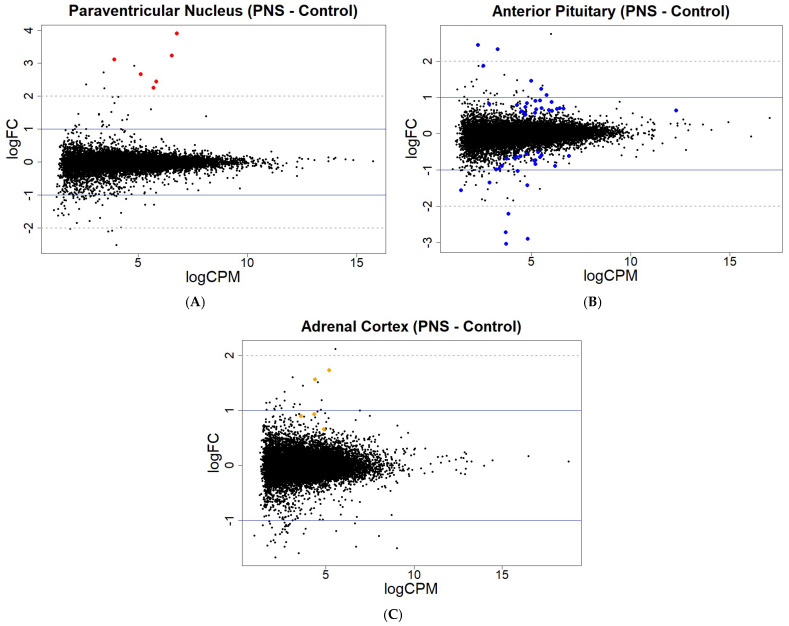
MA plots showing the relationship between the average concentration (logCPM) and fold-change (logFC) across the genes in each tissue. Each gene is represented by a dot with the differentially expressed genes (FDR < 0.15) colored in (**A**) red for the PVN, (**B**) blue for the PIT, and (**C**) orange for the AC. The blue solid lines represent a logFC ± 1 threshold, and the gray dotted lines represent a logFC ± 2 threshold.

For the gene expression at an FDR < 0.05, only one gene in the PVN was differentially expressed, while the PIT had the greater number of differentially expressed genes, with four up-regulated and five down-regulated (FDR < 0.05). The AC contained genes that were differentially expressed, with all four of the genes being up-regulated in the PNS relative to the Control cows. With reduced stringency, differential expression was seen for 6, 49, and 5 genes (FDR < 0.15) in the PVN, PIT, and AC, respectively, with most of the genes being up-regulated in the PNS group relative to the Control group (Figure 2; Supplementary Table S2). In the PIT, 24 of the differentially expressed genes (FDR < 0.15) were down-regulated in the PNS group compared to the Control. Overall, the portion of differentially methylated CpG sites out of all the sites analyzed, and the differentially expressed genes out of all the genes analyzed were low (<0.001% and <0.01%, respectively).

The analysis of the differentially methylated CpG sites (FDR < 0.05 and FDR < 0.15) with Ensembl BioMart (Ensembl Release 108) revealed whether these sites were located within either the promoter region or the exons or introns of gene body regions (Table 3). Reduced transcription and gene expression have been demonstrated with elevated DNA methylation within the promoter regions of a gene [42,43]. Methylation within gene body regions, such as introns and exons, has been positively correlated with transcription levels, with a hypothesized mechanism of blocking alternate promoters and regulatory regions to enhance the efficiency of transcription elongation [44]. Except for the PVN, there were greater amounts of differentially methylated CpG sites within the introns of gene body regions compared to the other regions. Also, the AC consistently showed more differentially methylated CpG sites within the promoter regions than the exons of gene body regions. There was no overlap of the genes associated with the differentially methylated CpG sites and the differentially expressed genes detected in each tissue of the stress axis, despite the known positive correlations between DNA methylation levels in transcribed regions and gene expression levels [45].

### 3.2. Gene Ontology Enrichment

**Figure 3:**
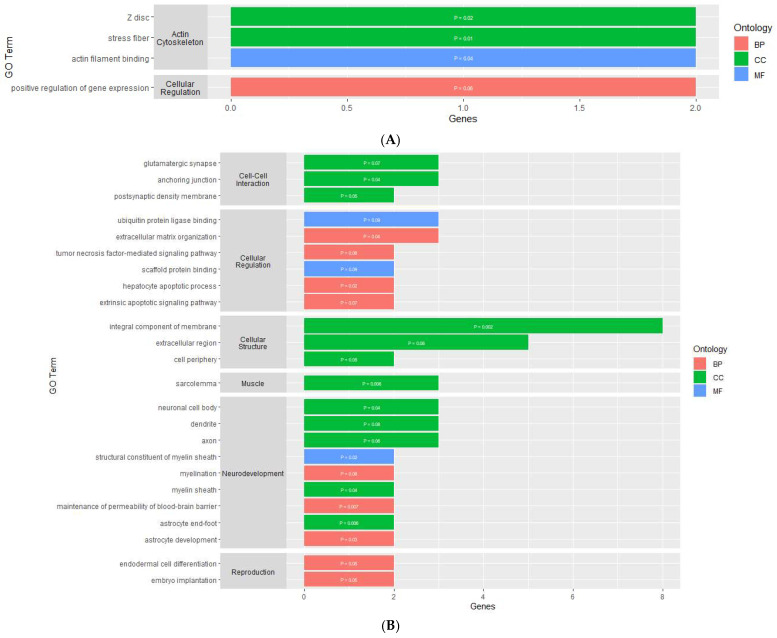

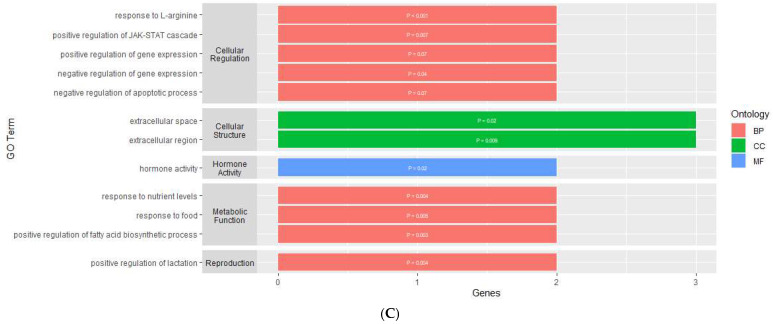
Gene ontology enrichment results of genes that are differentially expressed within the (**A**) PVN, (**B**) PIT, and (**C**) AC. *y*-axis contains GO terms and *x*-axis contains number of genes enriched within each GO term; *p*-value of each enriched term is within each individual bar.

The gene ontology analysis found the DMGs and DEGs were enriched in various gene ontology terms, categorized as either a biological process (BP), molecular function (MF), or cellular component (CC). For the PVN, the analysis using the DAVID Functional Annotation Tool resulted in four enriched GO terms for the DEGs and none for the DMGs (Supplementary Table S3). The GO terms for the DEGs were made up of one BP, one MF, and two CCs (Figure 3A). The cellular components and molecular function GO terms for the DEGs were related to the actin cytoskeleton, while the biological process GO term was related to cellular regulation through the positive regulation of gene expression.

**Figure 4:**
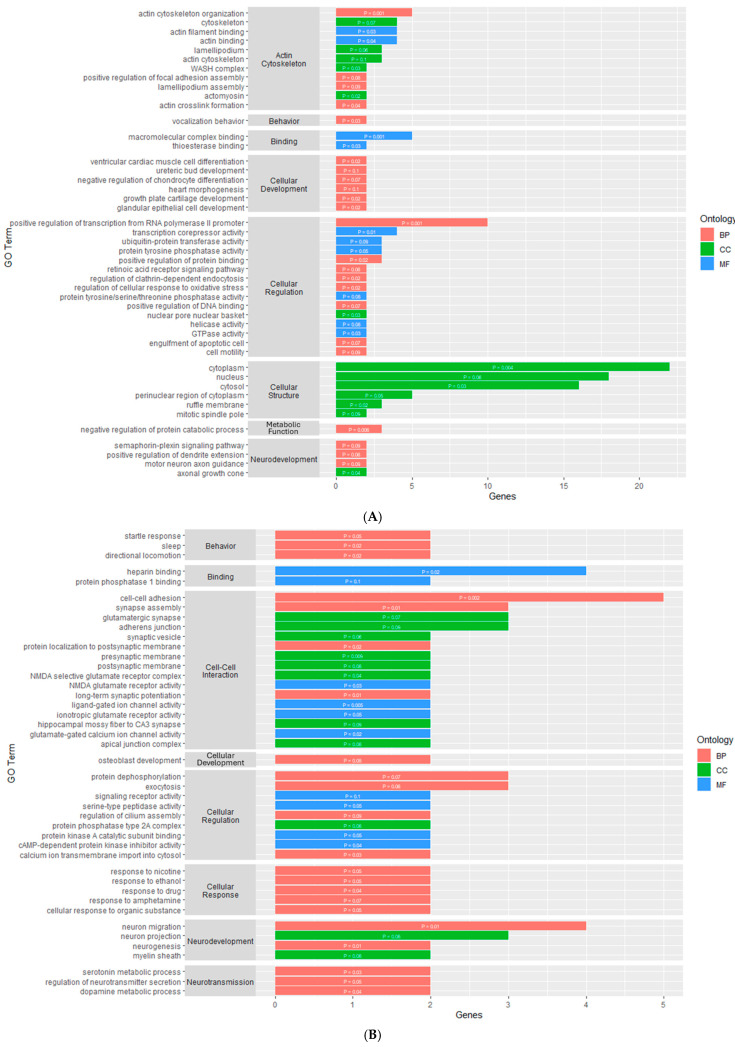
Gene ontology enrichment results for genes that are differentially methylated within the (**A**) PIT and (**B**) AC. *y*-axis contains GO terms and *x*-axis contains number of genes enriched within each GO term; *p*-value of each enriched term is within each individual bar.

In the PIT, there were 46 enriched GO terms for the DMGs and 24 enriched GO terms for the DEGs (Supplementary Table S4). The GO terms for the DMGs were made up of 23 BPs, 10 MFs, and 13 CCs (Figure 4A). Similar to the PVN, several GO terms were related to the organization and regulation of the actin cytoskeleton. There were also several GO terms associated with binding, cellular development, cellular structure, and cellular regulation. The GO terms for the DEGs were made up of 9 BPs, 12 CCs, and 3 MFs (Figure 3B). Nine of the GO terms for the DEGs were related to neurodevelopment and regulation. There were several GO terms associated with cellular structure, cell-to-cell interaction, and cellular regulation.

For the AC, the enrichment analysis resulted in 43 enriched GO terms for the DMGs and 12 enriched GO terms for the DEGs (Supplementary Table S5). The GO terms for the DMGs were made up of 22 BPs, 10 MFs, and 11 CCs (Figure 4B). Most of these GO terms were related to cellular processes, such as binding, cellular development, cell-to-cell interaction, cellular regulation, and cellular response. There were also four GO terms related to neurodevelopment and regulation, three terms related to synaptic transmission, and three terms related to behavior. The GO terms for the DEGs were made up of nine BPs, one MF, and two CCs (Figure 3C). One molecular function was related to hormone activity, one biological process was related to reproduction, and three biological processes were related to metabolic function. There were also several GO terms related to cellular structure and cellular regulation. The functional annotation analysis within the AC also resulted in two gene ontology clusters for the DMGs (Table 4). Annotation Cluster 1 had an enrichment score of 1.63 and contained GO terms made up of two MFs and two CCs that were related to cell-to-cell interaction and cellular regulation. Annotation Cluster 3 had an enrichment score of 0.61 and contained GO terms made up of three CCs that were related to cell structure and neurodevelopment.

### 3.3. KEGG Pathway Enrichment

**Figure 5:**
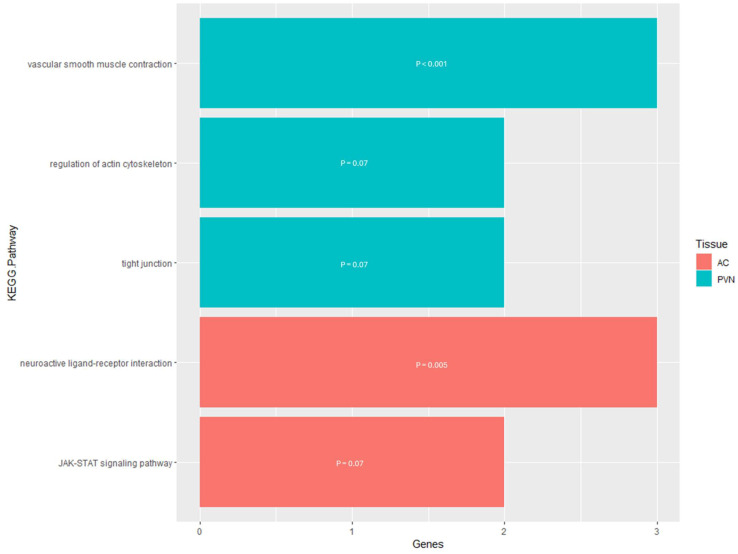
KEGG pathway enrichment results for the genes that are differentially expressed within the PVN and AC. The *y*-axis contains the KEGG pathway terms and the *x*-axis contains the number of genes enriched within each term; the *p*-value of each enriched term is within each individual bar.

The analysis using the DAVID Functional Annotation Tool for the results for the PVN resulted in three KEGG pathways for the DEGs that were related to the actin cytoskeleton and contraction (Supplementary Table S3). Two of the pathways had a direct relationship to the regulation of the actin cytoskeleton, while the third was related to smooth muscle contraction in the vasculature (Figure 5). The DEGs involved in the Regulation of the Actin Cytoskeleton KEGG pathway were concentrated toward the end of the pathway where actomyosin assembly contraction is regulated. Also, for the Tight Junction pathway, the DEGs were responsible for the portion of the pathway that regulates cell polarity in the regulation of the actin cytoskeleton. In terms of the Vascular Smooth Muscle Contraction KEGG pathway, the involved DEGs were end-products of the pathway working in conjunction to control contraction and relaxation of the smooth muscle.

**Figure 6:**
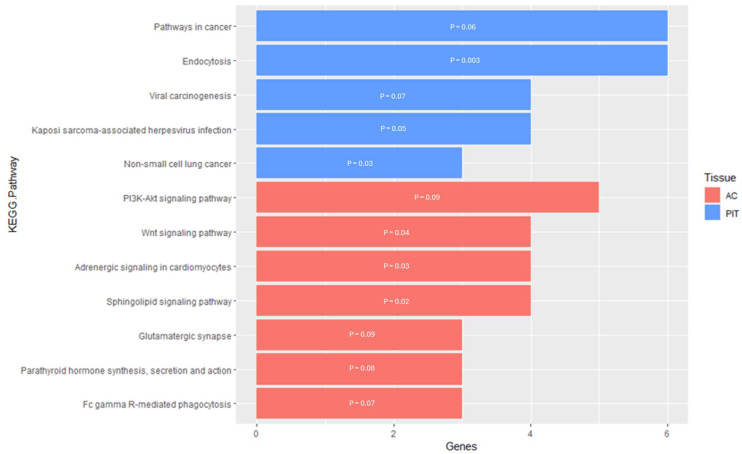
KEGG pathway enrichment results for genes that are differentially methylated within the PIT and AC. The *y*-axis contains the KEGG pathway terms and the *x*-axis contains the number of genes enriched within each term; the *p*-value of each enriched term is within each individual bar.

In the PIT, there were five enriched KEGG pathways for the DMGs (Supplementary Table S4). Four of the five pathways were related to cancer, whereas the last pathway was related to endocytosis (Figure 6). For the Endocytosis KEGG pathway, the DMGs were located within the pathway either at the plasma membrane or surrounding the endosomes within the cell. The functional annotation analysis within the PIT also resulted in two KEGG pathway clusters for the DMGs (Table 5). Annotation Cluster 1 had an enrichment score of 0.87 and contained six KEGG pathways. Of these pathways, three were related to cancer and/or a human-specific infection, while the other three were related to signaling pathways. Two of these signaling pathways (PI3K-Akt signaling pathway and Ras signaling pathway) are responsible for cellular regulation and the third (Chemokine signaling pathway) is responsible for the immune response to inflammation. Annotation Cluster 2 had an enrichment score of 0.66 and contained three KEGG pathways. One of the pathways was related to cancer, one was related to the actin cytoskeleton and cellular regulation (Focal Adhesion), and one was a signaling pathway (MAPK signaling pathway).

For the AC, the enrichment analysis resulted in seven enriched KEGG pathways for the DMGs and two enriched KEGG pathways for the DEGs (Supplementary Table S5). The pathways enriched for the DMGs were mostly related to cell signaling, which would lead to the regulation of apoptosis and cellular development and proliferation (Figure 6). However, three of the seven pathways were not directly related to cell signaling and instead were related to phagocytosis, synaptic transmission, and hormone activity. For the DEGs pathway enrichment results, one of the pathways was related to cell signaling with the Janus kinase/signal transducers and activators of transcription (JAK-STAT), while the other pathway was the Neuroactive Ligand–Receptor Interaction, responsible for regulating gene expression (Figure 5). The functional annotation analysis of the DMGs within the AC also resulted in one KEGG pathway cluster (Table 4). Annotation Cluster 2 had an enrichment score of 1.47 and contained three KEGG pathways. All three of these pathways were related to cell signaling, with the DMGs in the Sphingolipid signaling pathway being in the portion of the pathway responsible for apoptosis. Within the PI3K-Akt signaling pathway, the DMG was located either at the plasma membrane or the part of the pathway responsible for regulating the cell cycle. The KEGG pathway, Adrenergic Signaling in Cardiomyocytes, had the DMGs scattered throughout the signaling pathway.

## 4. Discussion

This project was designed to evaluate whether prenatal transportation stress affected DNAm and gene expression in the principal tissues of the hypothalamic–pituitary–adrenal stress axis of Brahman cows at 5 years of age. Transportation is one of several common livestock production practices that induce a stress response and can subsequently alter the metabolism, stress response, and immune system of offspring exposed to stressors in utero. The analysis of the results revealed no major effect on the genes coding for the classically recognized primary neuroendocrine products of the stress axis; however, numerous other genes within the stress axis tissues of the mature Brahman cows were affected by the prenatal transportation stress. These new findings regarding the concomitant assessment of DNAm and gene expression in the stress axis tissues of the Control and prenatally stressed cows are discussed.

### 4.1. Paraventricular Nucleus of the Hypothalamus

The PVN is a highly organized structure in the hypothalamus responsible for initiating the stress response, as well as for regulating cardiovascular homeostasis through the maintenance of blood pressure within a physiological range [46]. This is performed through the synthesis and secretion of various hormones, such as corticotrophin-releasing hormone (CRH), arginine vasopressin (AVP), and oxytocin (OXT). There were no DMGs or DEGs in our data corresponding to these hormones or the associated glucocorticoid receptors necessary for their regulation. Although fewer examples of DMGs and DEGs were detected in the PVN compared to the other tissues, these PVN genes were related to important processes in the brain, including the contraction of vascular smooth muscle cells and the maintenance and regulation of the actin cytoskeleton.

#### 4.1.1. PVN Actin Cytoskeleton

The cytoskeleton, located in all eukaryotic cells, is typically rich in actin, making it a dynamic scaffold responsible for maintaining cell shape, as well as regulating important cellular processes and neuronal polarity [47,48]. During the development of neurons, such as the magnocellular and parvocellular neurons within the PVN, the actin cytoskeleton plays a key role in axon guidance, neurite elongation, and synapse formation [49,50,51,52]. The genes in our data that were differentially expressed and involved with the actin cytoskeleton included *Acta2*, *Des*, *Myh11*, *Myl9*, and *Tagln*, all of which were up-regulated in the PNS cows compared to the Control cows (Supplementary Table S3). Various combinations of these genes were enriched in the Stress Fiber, Z Disc, and Actin Filament-Binding gene ontology terms, each of which are specifically related to actin filament. Actin filament, also known as F-actin, is one of the main regulators of neuronal polarity that can be driven by synaptic activity [48]. Similar to the enriched GO terms involved with F-actin, the enriched Regulation of the Actin Cytoskeleton and Tight Junction KEGG pathways in our results (*Myh11*, *Myl9*) are responsible for controlling cell polarity and regulating the actin cytoskeleton [53,54]. The interplay of tight junctions and the actin cytoskeleton influence cell polarity and thus epithelial permeability, which within the brain indicates involvement in the regulation of the blood–brain barrier [55,56]. Overall, the up-regulated genes in our data involved with these essential structures suggest that mature cows exposed to prenatal transportation stress have an increased regulation of cellular processes, neuronal polarity, and the blood–brain barrier, which may have been influenced by the development of PVN-specific neurons in utero. A future targeted immunostaining analysis of mature cow PVN tissue, as well as an analysis of fetal or neonatal PVN tissue, could provide insight into whether the differences in neurodevelopment between the PNS and Control cattle may have contributed to the increased expression of the genes identified in the mature PNS cows.

#### 4.1.2. Vascular Smooth Muscle Contraction

Another enriched KEGG pathway within the PVN, Vascular Smooth Muscle Contraction (*Acta2*, *Myh11*, and *Myl9*), plays a role in regulating intracranial blood pressure and cerebral blood flow through vasoconstrictors and myogenic contractions [57,58]. This pathway maintains the cerebral arterial diameter via actin polymerization and cytoskeletal dynamics [59,60,61]. Chasseigneaux et al. [62] determined that the expression of smooth muscle actin in rat brains was restricted to the vascular smooth muscle cells, and that several actin cytoskeleton-related genes (*Myh11*, *Acta2*, *Des*, and *Myl9*) were enriched in these cells compared to the mid-capillary pericytes. The up-regulation of these same genes in our data suggests an increase in cerebrovascular smooth muscle contraction, which could indicate an increased risk for high blood pressure in our PNS cows compared to Control cows [63,64]. The inhibition of *Acta2* expression results in impaired cell contraction and motility [65]; therefore, the up-regulation of *Acta2* in our data supports the proposition that there was increased cerebrovascular smooth muscle contraction within the PVN. Also, it is interesting to note that *Acta2*, along with *Mustn1*, were included in the enriched Positive Regulation of Gene Expression GO term; however, how these genes are involved in gene expression regulation requires further investigation.

### 4.2. Anterior Pituitary

Following the initiation of the stress response at the hypothalamic level, CRH travels to the anterior portion of the pituitary gland to stimulate the corticotroph cells to synthesize and secrete pro-opiomelanocortin (POMC), which is cleaved by prohormone convertase 1/3 to produce adrenocorticotropic hormone (ACTH) and β-endorphin [66,67]. However, POMC, ACTH, and β-endorphin, as well as other important components for continuing the stress response within the PIT (CRH receptor type I and II, glucocorticoid receptor, and AVP receptor), were neither differentially methylated nor differentially expressed in our data. The DMGs and DEGs in the PIT data were connected to the actin cytoskeleton, signal transduction, transcription, and neurodevelopmental processes, as discussed in the following sections (Section 4.2.1, Section 4.2.2, Section 4.2.3, Section 4.2.4, Section 4.2.5).

#### 4.2.1. Actin Cytoskeleton

Similar to the PVN, the DMGs and DEGs in our PIT data corresponded with several enriched GO and KEGG terms related to the actin cytoskeleton. Of the 19 DMGs and two DEGs involved, 5 DMGs (*Cdc42bpb*, *Capzb*, *Rac1*, *Flna*, and *Phactr1*) were found in three or more enriched terms that are related to the actin cytoskeleton’s organization, cell motility, and focal adhesion. All five of these DMGs were found in the Actin Cytoskeleton Organization GO term, which is a process responsible for the actin cytoskeletal structure’s assembly, arrangement, and disassembly. Actin exists in both monomeric (G-actin) and filamentous (F-actin) forms, and its ability to provide cell structure and support internal movements is regulated by actin-binding proteins [68,69,70]. *Phactr1* and *Flna* were found in the Actin-Binding and Actin Filament-Binding GO terms, respectively, while *Capzb* was found in both of these enriched terms. Each of these genes encodes for an actin-binding protein that plays a role in the organization of the actin cytoskeleton. *Capzb* encodes for the CapZβ protein that caps the barbed ends of actin filaments [71,72], *Phactr1* encodes for phosphatase and actin regulator 1 that are responsible for actin polymerization and cell motility [73,74], and *Flna* encodes for the filamin A protein that crosslinks actin filaments and anchors transmembrane proteins to the actin cytoskeleton [75,76]. Both *Capzb* and *Phactr1* were hypomethylated within the gene body regions, suggesting the decreased expression of these genes in the PNS cows relative to the Controls; however, *Flna* was hypermethylated within a gene body region indicating increased gene expression. The reduced expression of CapZβ has been shown to result in cell death and tissue degeneration due to the abnormal accumulation of actin from the reduced capping of the F-actin ends [77,78], while the *Phactr1* gene has been shown to decrease cell motility and morphology with its reduced expression, particularly in neuronal cells during development [79,80]. Several studies have shown how the increased expression of FLNA is associated with neurological disorders, tumor development, and decreased survival rates [81,82,83]. Though we did not identify any phenotypic differences between the treatment groups for the pituitary gland, it is likely that the altered expression of these genes in the PNS cows could be indicative of the reduced functionality of the actin cytoskeleton within the anterior pituitary.

#### 4.2.2. Cell Motility

An important function of the actin cytoskeleton is cell motility, which aids in various cell developmental processes, such as whole-cell migration, intracellular motility, axon guidance, tissue regeneration, and embryological development [84,85]. The actin cytoskeleton’s control of cell motility is achieved by actin polymerization, which is achieved through the synergistic work of multiple cytoskeletal functions and components, such as GTPase activity, actomyosin, lamellipodia, actin crosslink formation, and focal adhesions [85,86,87]. All five of the aforementioned actin cytoskeleton-related DMGs work together in various combinations to regulate the processes needed to promote cell motility via the actin cytoskeleton. The genes that had hypomethylation within the gene body region (*Rac1*, *Capzb*, and *Phactr1*) were related to the Cell Motility, GTPase Activity, Lamellipodium Assembly, and Positive Regulation of Focal Adhesion Assembly GO terms, while the gene body region’s hypermethylated genes (*Cdc42bpb*, *Flna*) were related to Actomyosin and Actin Crosslink Formation. The enriched GO terms that included both hypo- and hypermethylated genes included Semaphorin–Plexin signaling pathway and Lamellipodium, as well as the Focal Adhesion KEGG pathway.

The *Phactr1* gene found in the Cell Motility term is related to actin polymerization, where the down-regulation of this gene has been shown to reduce cell migration and reorganization of the actin cytoskeleton [80,88]. The Cell Motility term also contained *Rac1*, which is listed in several of the Cell Motility-related GO and KEGG terms, including GTPase Activity, Semaphorin–Plexin signaling pathway, Lamellipodium, Lamellipodium Assembly, Focal Adhesion, and Positive Regulation of Focal Adhesion Assembly. The role of *Rac1* in each of these terms is to encode the Rac1 protein that is one of three well-known Rho GTPases responsible for transducing cellular signals, such as through the Semaphorin–Plexin signaling pathway [89], to regulate cell polarity, morphogenesis, migration, and apoptosis [90,91]. Within the leading edge of a migrating cell, Rac1 enhances focal adhesion formation [92,93], while Rac1 localized in the lamellipodia regulates F-actin polymerization through the activation and mediation of multiple protein complexes [91,94]. The decreased expression of *Rac1* leads to reduced cell motility and disrupted cell adhesion [92]. The formation of lamellipodia where Rac1 is localized is reliant on the rapid capping of the barbed ends of F-actin controlled by the capping proteins, such as CapZβ [72,95,96]. The reduced expression of the gene responsible for CapZβ production has been shown to not only accumulate abnormal amounts of actin, but also cause the loss of lamellipodia, resulting in diminished cell motility and ultimately cell death and tissue degeneration [78,97].

Lamellipodium formation, along with filopodia protrusion, is also dependent on actin filament crosslinking regulated by actin cross-linkers, such as α-actinin and filamin A [98]. In our data, the gene that encodes for filamin A (*Flna*) was hypermethylated, potentially compensating for the hypomethylation of *Capzb*, though further analysis of the actin filament network would need to be conducted to determine any phenotypic differences between the PNS and Control’s anterior pituitary tissues. In addition to actin crosslink formation, filamin A regulates the cell–extracellular matrix interaction [99,100] and acts as a scaffolding protein to anchor transmembrane integrins, such as plexin in the Semaphorin–Plexin signaling pathway [101]. In contrast to Rac1, filamin A is responsible for the disassembly of focal adhesions at the leading edge of a migrating cell to promote cell spreading and forward movement during cell motility; thus, the hypermethylation of *Flna* and the hypomethylation of *Rac1* indicate the dysregulation of focal adhesion assembly within the PIT of our PNS cows, potentially contributing to impaired cell motility [102].

Cdc42 is another well-known Rho GTPase that induces filopodia extension, as well as working in conjunction with Rac1 to activate Rac-dependent lamellipodium protrusion in order to promote the polymerization mechanism of cell motility [103,104]. Its functions are regulated by various effector proteins, including myotonic dystrophy kinase-related Cdc42-binding kinase β (MRCKβ), which also work to promote the motor mechanism of cell motility via actomyosin contraction [105]. MRCKβ, encoded by *Cdc42bpb*, was found in both the Lamellipodium and Actomyosin GO terms in our data. The elevated expression of this gene has been shown to increase cell migration through actomyosin contractility [106], though its overexpression has also been discovered in some cancers, suggesting a role in tumor progression [107]. Our data suggest the potential down-regulation of the genes related to controlling cell motility via actin polymerization. However, the hypermethylation of *Cdc42bpb* may provide a mechanism for overcoming diminished lamellipodia and focal adhesion development through the motor actomyosin mechanism, since cell migration could be achieved by one or both of these mechanisms [108]. Overall, the hypo- and hypermethylation of these five genes suggests that the actin cytoskeleton’s organization and cell motility were impaired within the anterior pituitary of the PNS cows, which could indicate a reduced ability for tissue regeneration and increased pituitary-related morbidity compared to the Control cows.

#### 4.2.3. Anterior Pituitary Signal Transduction

The analysis of the DMGs revealed four genes (*Rac1*, *Gnb1*, *Egfr*, and *Pik3r6*) enriched in several KEGG pathways related to cell signaling, such as the Ras signaling pathway, MAPK signaling pathway, PI3K-Akt signaling pathway, and Chemokine signaling pathway. For the genes involved, two were differentially methylated within the gene body regions (hypermethylated: *Pik3r6*; hypomethylated: *Rac1*) and two were differentially methylated within the promoter regions (hypermethylated: *Egfr*; hypomethylated: *Gnb1*). Based on the location of methylation in these genes, we will assume that *Rac1* and *Egfr* expression could potentially be down-regulated, while *Gnb1* and *Pik3r6* expression could potentially be up-regulated, though there was no overlap of genes that were differentially methylated in our DEGs data.

These four enriched signaling pathways have been found to be closely interrelated, in that certain downstream components, such as Ras and Rac, are involved in multiple signaling pathways which elicit several cellular effects. One of these effects is the regulation of apoptosis, either through pro- or anti-apoptotic signaling, in which the DMGs from our data set play a particular role [109,110]. Signaling via a tyrosine receptor, such as the epidermal growth factor receptor (EGFR) encoded by *Egfr*, typically results in cellular growth, survival, proliferation, and differentiation when the signal cascade goes through the PI3K-Akt and classical MAPK pathways. However, the EGFR also plays an anti-apoptotic role when cell signaling follows the JNK/p38 MAPK pathway, suggesting that the EGFR is important for maintaining the delicate balance between pro- and anti-apoptotic signaling [111,112,113]. Rac1, which, as previously mentioned, is a Rho GTPase protein responsible for transducing cellular signals, is an essential downstream component of the MAPK, PI3K-Akt, and Chemokine signaling pathways that is regulated via the activation of the Ras signaling pathway. Within the PI3K-Akt signaling pathway it plays an anti-apoptotic role by stimulating PI3K Class I_A_ [114,115,116], while in the MAPK pathway it plays a pro-apoptotic role through JNK/p38 signaling [117,118]. Thus, similar to the EGFR, Rac1 plays an important role in maintaining balance in apoptosis regulation. The dual role of both genes in apoptosis indicates that their potential down-regulation would not result in a drastic change in cell survival; however, within our data, there was also evidence of the up-regulation of *Gnb1* and *Pik3r6*, which are also important components of several signaling pathways responsible for cell survival regulation.

The G protein subunit β1 (Gβ), encoded by *Gnb1*, is an essential component of G protein-coupled receptors (GPCRs) that forms a heterotrimer with a Gα and Gγ. The activation of GPCRs result in Gα separating from Gβ/Gγ, and each unit continues signal transduction to elicit their own cellular responses within the Ras, PI3K-Akt, and Chemokine signaling pathways [119,120,121]. The Gβ/Gγ heterodimer stimulates PI3K Class I_B_, which also plays an anti-apoptotic role, like PI3K Class I_A_. Class I_B_ PI3Ks are regulated by several subunits, such as phosphoinositide-3-kinase regulatory subunit 6 (p87) encoded by *Pik3r6*, which drive the activation of the catalytic subunit γ of PI3K to produce the lipid second messenger PIP3 and further transduce the cell signal [116,122]. The up-regulation of both of these genes has been associated with increased cell survival, possibly playing a role in promoting tumor progression [123,124,125]. These results indicate that apoptosis within the anterior pituitary was inhibited in the mature cows that were exposed to prenatal transportation, suggesting that stress experienced prenatally leads to long-term effects in cell turnover. This is supported by studies in rodents that have demonstrated how prenatal stress inhibits apoptosis in different tissues, particularly the hippocampus–hypothalamus–pituitary axis [126,127].

The DEGs enrichment analysis identified three signaling pathway-related GO terms: the (1) Extrinsic Apoptotic signaling pathway, (2) Tumor Necrosis Factor-mediated signaling pathway, and (3) Scaffold Protein Binding. Each of these signaling pathways include the up-regulated *Krt8* and *Krt18* genes. These two genes encode for keratin proteins, keratin 8 (KRT8) and keratin 18 (KRT18), respectively, that dimerize (KRT8/KRT18) to form the intermediate filament scaffold that maintains cell shape, protects against mechanical stress, and regulates cell survival signaling pathways. These keratin proteins are ubiquitously expressed in the epithelial cells, including the endocrine cells of the anterior pituitary [128,129,130]. Exposure to stress increases the expression of KRT8/KRT18 [131]. This increase in expression is negatively associated with the apoptotic signaling pathways, and ultimately results in tumor progression [132,133,134,135,136]. Though our DMGs data did not overlap with our DEGs data, the up-regulation of these two genes coincides with the literature and indirectly confirms our suspicion that prenatal stress impairs cytoskeletal organization and multiple cellular processes, potentially increasing pituitary-related morbidity in our mature PNS cows.

#### 4.2.4. Transcription

In addition to the signaling pathways, there were five enriched GO terms related to the regulation and control of transcription, the important first step in gene expression. Four of the five genes found in various combinations in these GO terms were differentially methylated within the gene body regions (hypermethylated: *Hipk2*, *Irf2bpl*, and *Rara*; hypomethylated: *Prkn*), while *Rarb* was hypermethylated in the promoter region. The Positive Regulation of DNA Binding, Transcription Corepressor Activity, and Positive Regulation of Transcription from RNA Polymerase II Promoter were the GO terms that included both *Hipk2* and *Prkn*, each of which plays an important role in regulating the transcription factors responsible for apoptosis. The enzyme homeodomain-interacting protein kinase 2 (HIPK2), encoded by *Hipk2*, is located predominantly within the cell nucleus and has a dual role in transcription regulation as a both a co-activator and co-repressor. When interacting with NK homeodomain transcription factors, HIPK2 acts as a co-repressor by increasing their DNA binding affinity and repressing transcription [137], yet it also promotes transcription by acting as a co-activator of apoptotic transcription factors through the phosphorylation of the p53 protein [138,139]. The affinity of parkin, a ligase encoded by *Prkn*, to bind DNA reduces the transcription of p53, which enhances cell survival by preventing its apoptotic activity in conjunction with HIPK2. Parkin also enhances cell survival and protects cells from stress-induced apoptosis through the NF-κB signaling pathway [140,141]. Contrary to our initial speculation, the differential methylation of these genes in the PIT data appears to have impaired cell survival in the PNS cows, with the potential up-regulation of *Hipk2* and down-regulation of *Prkn*. It is possible, since the anterior pituitary contains several hormone-producing cell types, that these contrasting results were produced by cell types other than the corticotrophs, though further analysis with single-cell sequencing methods would be needed for verification. In response to oxidative stress, which plays an important role in signal transduction and transcription, HIPK2 maintains its function of facilitating apoptosis and inhibiting tumor growth [138,142]. Additionally, parkin, a component of the Regulation of Cellular Response to Oxidative Stress GO term, also works as a critical tumor suppressor by protecting against oxidative stress [143]. Though the function of parkin as a tumor suppressor was potentially repressed in the PNS cows, the suppressive actions of HIPK2 on tumor growth as an apoptotic factor was potentially overexpressed. This could suggest that the differential methylation of *Hipk2* may be a corrective measure for minimizing the negative effects of prenatal stress within the anterior pituitary.

Other DMGs that had a dual role in regulating transcription were *Rara* and *Rarb*, which were found in the Positive Regulation of Transcription from RNA Polymerase II Promoter and Retinoic Acid Receptor signaling pathway GO terms. Both genes encode for retinoic acid receptors, which are ligand-dependent DNA-binding transcriptional regulators. Both isoforms have redundant and specific roles in regulating transcription controlled by the motif of their target genes, with retinoic acid receptor (RAR) α being a strong repressor of target gene expression and RARβ limiting cell growth by regulating gene expression [144,145]. With the potential up-regulation of RARα and the down-regulation of RARβ, it appears that the specific roles of these receptors in regulating transcription were affected, though it is unclear what those specific roles could entail. The expression of these genes is under epigenetic regulation by promoter methylation, where RARβ promoter hypermethylation has been shown to increase the onset and progression of cancer [146,147,148], which correlates with the methylation status of our signaling pathway DMGs that indicated an increased risk of pituitary-related morbidity in the mature PNS cows.

The fifth DMG associated with the Positive Regulation of Transcription from RNA Polymerase II Promoter and Transcription Corepressor Activity GO terms, *Irf2bpl*, is a known transcription factor with a biological function that is still not fully understood, though mutations in this gene have manifested as neurological problems [149]. Within humans and rodents this gene is also known as EAP1 (enhanced at puberty 1), a factor that may play a role in regulating female reproductive function by transactivating the genes within the hypothalamus responsible for controlling the onset of puberty and regulating estrous cyclicity [150]. However, this alternate name is not present within the Ensemble Release 98 *B. taurus* reference genome used for this study, and the expression of this gene within the hypothalamic–pituitary–gonadal axis has not been explored in cattle. If this gene is required for enhancing reproductive function within cattle, then its potential up-regulation in our mature PNS cows does not coincide with the literature that has repeatedly demonstrated that stressors experienced prenatally negatively impact reproductive function in cattle [151,152]. It could be, since these cows were kept within the herd instead of being culled until they were euthanized at 5 years of age, that the prenatal transportation stress did not have as great of a negative impact on their reproductive function and instead enhanced this transcription factor to prevent impaired reproductive ability due to stress exposure. Further research into the expression of this gene within both the hypothalamus and anterior pituitary is needed to elicit its role in the reproductive function in cattle.

Another important GO term related to transcription that was enriched was Helicase Activity. Though the genes enriched in this term were not found in other relevant GO terms, it is interesting to note that *Ighmbp2*, which encodes for a helicase responsible for separating DNA strands [153,154], was hypomethylated in a gene body region, and *Zranb3*, which encodes a zinc finger responsible for DNA rewinding [155,156], was hypermethylated in a gene body region. This possibly means that helicase activity was down-regulated and DNA annealing activity was up-regulated in the anterior pituitary, preventing the transcription machinery from binding the DNA strands. Most of these genes have a dual role in transcription, so it is difficult to determine which role is either inhibited or promoted through the differential methylation of each gene. Based on the results indicating the inhibition of helicase activity, it can be assumed that transcription overall was repressed in the mature cows exposed to prenatal transportation, with the potential exception of *Irf2bpl* and *Hipk2* activity.

#### 4.2.5. Neurodevelopment and Glutamatergic Synapses

The glandular anterior pituitary is not heavy with nervous tissue like the posterior pituitary; however, studies have shown that it is innervated by both autonomic and hypothalamic nerve fibers [157,158,159]. For cells and organisms to respond to extrinsic and intrinsic factors, neurons are required to act as messengers by releasing chemical signals or passing electrical impulses through synapses from one neuron to either a target cell or another neuron [160,161]. Thus, the proper development of neurons, astrocytes, and synapses is essential for proper physiological function. In our PIT data, there were 12 neural- and two synapse-related enriched GO terms, with DMGs only found in the neural-related terms and the DEGs found in both the neural- and synapse-related terms. It is interesting to note that three out of five DMGs and 11 out of 14 DEGs related to neural and synapse GO terms were hypomethylated within the gene body regions or down-regulated, respectively. This means that there was potentially an overall decrease in the expression of genes responsible for nervous tissue components and neurodevelopment within the anterior pituitary of our PNS cows compared to the Controls.

The enriched GO terms included neuron structures (DEGs: Neuronal Cell Body, Axon, Dendrite, and Myelin Sheath), as well as the biological processes and molecular functions required for their formation (DMGs: Axonal Growth Cone, Motor Neuron Axon Guidance, Positive Regulation of Dendrite Extension; DEGs: Myelination, Structural Constituent of Myelin Sheath). For the DEGs, *Mbp* and *Plp1* were down-regulated and found in four or more terms associated with myelination and neuronal cell body and astrocyte development, while for the DMGs, only one gene (*Kif5c*) was found in more than one GO term, which was associated with axon-specific development. The interplay of each of these structures is important in facilitating synaptic transmission, where the myelination of axons enables rapid impulse conduction and maintains neuronal plasticity, while astrocytes control transmission through their close association with synapses [162,163]. The interaction of the proteins encoded by *Mbp*, myelin basic protein, and *Plp1*, proteolipid protein 1, is responsible for regulating myelination to form and maintain the myelin sheath. The dysregulation of these genes decreases myelination and has been associated with neurological disorders [164,165,166,167]. This suggests that the presence of a myelin sheath surrounding the neurons and synaptic plasticity within the anterior pituitary of the PNS cows may have been reduced, resulting in prolonged conduction along the axons in relation to the Control cows’ neurons. Axon development and growth, especially those of the motor neurons, have been shown to be reliant on the expression of *Kif5c*, which produces a kinesin heavy chain subunit protein responsible for organelle transport and synaptic transmission. The regulation of this transport plays an important role in brain development, plasticity, and survival [168,169]. This gene was hypermethylated within a gene body region, potentially up-regulating its expression within the anterior pituitary of the PNS cows, suggesting that while myelination may have been impaired, axonal maintenance was not.

There were only two synapse-related GO terms (Glutamatergic Synapse, Postsynaptic Density Membrane), both of which contained the up-regulated *Neto1* and down-regulated *Plppr4*. Within the anterior pituitary glutamate, a major excitatory neurotransmitter is able to elicit its functions through the glutamatergic synapse located between the hypothalamic neurons and the hormone-producing cells, such as corticotrophs [170,171,172]. Within the hippocampus, neuropilin and tolloid-like 1 (NETO1), encoded by *Neto1*, was found to be responsible for guiding the development of this synapse by regulating the kainate receptors within the axon, revealing its essential role in spatial learning and memory [173,174]. The altered expression of this gene has been implicated in various cancers, with its overexpression in ovarian cancer, resulting in increased cell migration and invasion, indicating an increased risk of pituitary-related morbidity in the PNS cows relative to the Controls [175]. The phospholipid phosphatase encoded by *Plppr4* regulates cells’ functions through the dephosphorylation of various lipid mediators and indirectly regulates glutamatergic synapse transmission as a postsynaptic density membrane protein [176,177]. The loss of this gene in mice altered their stress-related behavior due to increased glutamate release leading to neuronal hyperexcitability, a pathophysiological feature in certain neurological disorders [177].

### 4.3. Adrenal Cortex

As the distal component of the stress axis, the adrenal gland plays a major role in mediating the stress response. The cortex of the adrenal glands is primarily responsible for producing glucocorticoids, such as cortisol, to initiate various effects within the reproductive, immune, digestive, cardiovascular, and respiratory systems via gluconeogenesis and anti-inflammatory pathways [4,178]. Various hormones and proteins must be synthesized and secreted to achieve these effects, including the ACTH receptor, P450scc, StAR Protein, 11β-hydroxylase, and cortisol, though the corresponding genes were not found within the DMGs or DEGs of our AC data set. The genes that were differentially methylated or differentially expressed were related to important processes within the adrenal cortex, such as synaptic transmission, signal transduction, post-translational modifications, and immune responses.

#### 4.3.1. Synaptic Transmission and Glutamatergic Synapse

Several DMGs in the AC data were enriched with 28 GO and KEGG terms related to synaptic transmission. Of the 25 DMGs involved, 3 genes (*Grin2a*, *Grik4*, and *Kirrel3*) were found in three or more enriched terms related to neuron and synapse formation (eight GO terms), transmission (five GO terms), and glutamatergic synapse function (one KEGG and seven GO terms). Though the adrenal cortex is principally a glandular organ made up of epithelial cells, it is also innervated with adrenomedullary chromaffin cells and thoracic splanchnic nerves, which help mediate the adrenocortical response to stress and the diurnal rhythm of glucocorticoid production through non-ACTH mechanisms [179,180,181,182]. The cross-talk between the adrenal cortex and medulla facilitates the paracrine action of adrenal catecholamines and steroids; thus, the presence of several neural- and synaptic-related enriched terms in our AC data set is not surprising. All three DMGs had differential methylation within the gene body regions, with *Grin2a* and *Kirrel3* being hypomethylated and *Grik4* being hypermethylated. Both *Grin2a* and *Grik4* encode for glutamate ionotropic receptors, GluN2A, an NMDA receptor subunit type 2A, and GluK4, a kainate receptor subunit type 4, respectively. With both being a part of the glutamate-gated ion channel family, the binding of agonists activates the ligand-gated ion channels responsible for synaptic transmission and regulating long-term potentiation [183,184,185]. Though not a receptor, *Kirrel3* encodes for a transmembrane protein known as nephrin-like protein 2 (NEPH2), which is a synaptic cell adhesion molecule responsible for synaptic formation and transmission, as well as mediating cell migration [186,187].

Only *Grin2a* and *Kirrel3* were found in the GO terms related to neuron structure and formation (Neurogenesis, Neuron Projection, Neuron Migration, and Cell-Cell Adhesion), while all three DMGs were included in the GO terms related to synapse structure and formation (Synapse Assembly, Presynaptic Membrane, Postsynaptic Membrane, and Synaptic Vesicle). Similar to the anterior pituitary, the proper development of neurons, their projections, and synaptic membranes is essential, yet the potential down-regulation of *Grin2a* and *Kirrel3* suggests impaired neuron and synapse development within the adrenal cortex of the mature PNS cows. There have not been studies conducted to determine how the down-regulation of these two genes impacts the adrenal gland and organism response to stress; however, the dysregulation of these genes within the brain have been shown to result in decreased cell survival, proliferation, and migration, as well as an increased risk of developing neurological disorders [188,189,190]. In contrast to *Grin2a* and *Kirrel3*, *Grik4* was potentially up-regulated in the PNS cows relative to the Controls. The expression of this gene within the brain was shown to contribute to heightened anxiety and depressive behavior, as well as kainate-induced neuronal cell death, indicating that the up-regulation of this gene could enhance the degeneration of neurons within the adrenal cortex by kainate [191,192,193].

In addition to neuron and synapse formation, *Grin2a* was found in a majority of the synapse transmission-related GO and KEGG terms (13), with *Grik4* being included in the Signaling Receptor Activity, Glutamatergic Synapse, and Ligand-Gated Ion Channel Activity terms alongside *Grin2a*. Similar to the anterior pituitary, the glutamatergic synapse plays an important role between the nerve fibers and stress-hormone-producing cells found within the adrenal cortex. It has been suggested that glutamate receptors are partly responsible for releasing catecholamines from bovine chromaffin cells and regulating the long-term effects of stress on adrenal function [194,195,196]. Though the activity of ionotropic glutamate receptors within the adrenal gland has not been extensively researched, the blockage of NMDA-specific receptor channels, such as GluN2A, in rat adrenal glands led to an increase in AP-1 transcription factor activity, which is responsible for regulating gene expression in response to various stimuli, including stress [197]. It is still not clear the role that each of these ionotropic glutamate receptors plays within the adrenal cortex; however, the potential down-regulation of *Grin2a* and up-regulation of *Grik4* in our PNS cows suggests either a differential or compensatory role in regulating the stress response via glutamate-controlled mechanisms.

It is also important to note that the DMG *Crhr2*, which encodes for CRH receptor type 2 (CRHR2), was included in the Long-Term Synaptic Potentiation GO term with *Grin2a*, though this gene, which was hypermethylated within the promoter region, was not included in any other enriched GO or KEGG terms in our AC data set. The activity of CRH and its receptors is well known within the anterior pituitary, though CRH has been shown to directly stimulate glucocorticoid and catecholamine release from the adrenal gland [198,199]. The role of CRHR2 in strengthening synapses through long-term potentiation within the adrenal gland has not yet been investigated; nevertheless, CRH and its receptors have been localized within the synapses of the central and peripheral nervous systems, where there play a neuroregulatory role affecting synaptic transmission and plasticity [199,200]. The potential down-regulation of this gene within the mature PNS cows relative to the Control cows could indicate an impaired adrenal cortex stress axis function, though no other stress axis-related genes were found to be differentially methylated or expressed. The involvement of *Grin2a* in most of the neuron- and synaptic-related enriched terms indicates its essential role in regulating neuron development and synaptic function, warranting further investigation into its specific role within the adrenal cortex during development and in mediating responses to stress.

#### 4.3.2. Adrenal Cortex Signal Transduction

The analysis of the DMGs revealed three genes (*Bcl2*, *Ppp2r2c*, and *Ppp2r3b*) enriched in three of the four KEGG pathways related to cell signaling, such as the Sphingolipid signaling pathway, PI3K-Akt signaling pathway, and Adrenergic Signaling in Cardiomyocytes. All three of the genes involved were hypomethylated within the gene body regions, suggesting these genes could be down-regulated in the PNS cows compared to the Control cows, though, similar to the PIT, there was no overlap of the genes that were differentially methylated in our DEGs data. Each of these pathways are essential for regulating various cellular processes, such as apoptosis [201,202,203], which is an important process within the adrenal cortex as it continuously undergoes cell renewal and remodeling [204,205]. B-cell lymphoma 2, also known as BCL2 apoptosis regulator (Bcl-2), is encoded by *Bcl2* and found in the outer mitochondrial membrane where it promotes cell survival through the inhibition of pro-apoptotic protein action in multiple signaling pathways [206,207,208,209]. The down-regulation of this gene has thus been associated with decreased cell survival through both apoptotic and autophagic mechanisms [210,211], which could mean there was an increased turnover of adrenocortical cells in the PNS cows, potentially indicating the chronic activation of cells in response to the prenatal transportation stress stimulus. The increased proliferation, migration, and turnover of adrenocortical cells in response to acute stress has been demonstrated in different rodent studies [212,213]; however, the long-term impact on adrenocortical cell turnover after exposure to prenatal stress has not been explored.

Both *Ppp2r2c* and *Ppp2r3b* encode regulatory B subunits of protein phosphatase 2A (PP2A), specifically PR55γ and PR48/PR70, respectively [214,215]. The B subunit is what determines PP2A substrate specificity and activity, though the overall function of PP2A is a tumor suppressor through its regulation of cell proliferation, migration, and survival in various signaling pathways [216,217]. As a serine/threonine phosphatase, PP2A can directly interact with serine/threonine protein kinases, such as Akt, and cell survival effectors, such as Bcl-2, to restrain cell proliferation and induce apoptosis. Within the Sphingolipid signaling pathway, PP2A is activated by ceramide, a Sphingolipid that binds and disassociates the inhibitor SET from PP2A, to increase its tumor-suppressive activity [218,219]. In general, the inhibition of PP2A activity, via the down-regulation of its regulatory B subunits, would promote tumorigenesis due to its inability to inhibit Akt and Bcl-2 anti-apoptotic activity. In contrast to this notion, the down-regulation of PR55γ revealed a pro-apoptotic role due to its ability to bind and inhibit c-Src, a kinase responsible for activating the JNK apoptotic signaling pathway [220,221]. The pro- and anti-apoptotic consequence of potential *Ppp2r2c* and *Ppp2r3b* down-regulation, respectively, presents a contrasting effect in the adrenal cortex maintenance of the cows exposed to prenatal transportation stress. Yet, with the combined potential down-regulation of *Bcl2* in our AC data set, it is likely that cell turnover within the adrenal cortex was increased in the stressed cows relative to the Controls. Within the cardiomyocytes, PP2A is activated by β-adrenergic receptors, which phosphorylate the PR56δ regulatory B subunit after stimulation [222]. The regulation of PP2A by adrenergic signaling has not been explored within the adrenal gland, and it has yet to be shown to mediate the other regulatory subunits of PP2A, such as the two DMGs in our AC data set. Thus, though *Ppp2r2c* and *Ppp2r3b* were included in the Adrenergic Signaling in Cardiomyocytes KEGG pathway, it is not clear whether they play a role in adrenergic signaling and what their potential down-regulation would mean in the PNS cows relative to the Controls regarding catecholamine signaling within the adrenal cortex.

In addition to the above signaling pathways, there were two enriched GO terms, Protein Kinase A Catalytic Subunit Binding and cAMP-Dependent Protein Kinase Inhibitor Activity, that are both associated with the cyclic AMP (cAMP) signaling pathway. There was one common gene included in each of these terms that was hypermethylated within the gene body region, *Prkar1b*, which encodes for the type Iβ regulatory subunit (RIβ) of protein kinase A (PKA). Within the adrenal cortex, PKA is responsible for inducing steroidogenesis through the cAMP pathway, and so the dysregulation of this pathway can lead to adrenal insufficiency [223,224]. Two regulatory subunits and two catalytic subunits make up the inactive form of PKA that is dependent on cAMP interaction for the dissociation and activation of the catalytic subunits, which mediate protein activity via phosphorylation [225,226]. The activity of PKA is negatively regulated in multiple ways, such as through a negative feedback loop with the proteins that activate PKA phosphorylates, as well as the inhibition of catalytic subunit activity by PDK1 and the increased expression of regulatory subunits. It has been demonstrated that the up-regulation of regulatory subunits, such as RIβ, results in an increased regulatory–catalytic subunit complex interaction regardless of the cAMP concentration and decreased distance of the catalytic subunit action before being recaptured by the regulatory subunits, ultimately decreasing PKA activity [224,226]. Decreased PKA activity impacts various cellular functions, such as mitochondria-dependent apoptosis, since it is unable to modulate protein activity by phosphorylation. Ilouz et al. [227] demonstrated that RIβ is localized to the mitochondria, indicating its unique role in mitochondrial function compared to other PKA regulatory subunits, and mitochondrial cAMP signaling via PKA has shown an anti-apoptotic role. Though the specific relationship between PKA activation and Bcl-2 function is largely unknown, the phosphorylation of Bcl-2 is associated with the inhibition of apoptosis; thus, it is possible that PKA could play a role in promoting Bcl-2 function via phosphorylation, as decreased PKA activity has been shown to increase apoptosis [206,228]. Therefore, the potential down-regulation of *Bcl2* mentioned earlier, and the potential up-regulation of *Prkar1b* in the PNS cows could indicate increased apoptosis within the adrenal cortex, as well as adrenal insufficiency caused by decreased PKA activity.

Within the AC DMGs, the Wnt signaling pathway and Parathyroid Hormone Synthesis, Secretion, and Action KEGG pathways were enriched, with one common gene (*Lrp5*) between the two terms that encodes for low-density lipoprotein receptor-related protein 5 (LRP5), which acts as a co-receptor required for canonical Wnt signaling [229]. Signaling via this pathway is responsible for promoting the differentiation of the adrenal cortex outer zona glomerulosa, as well as the production of aldosterone from this zonation. The Wnt signaling pathway is one of the most mutated pathways within adrenocortical tumors due to its complexity and plethora of receptor types [230,231]. Nevertheless, due to *Lrp5* being both hypo- and hypermethylated within the gene body region, it is not clear whether Wnt signaling was modified in the mature cows exposed to prenatal stress relative to the Control cows. This gene was also included in the parathyroid hormone (PTH) KEGG pathway that promotes aldosterone secretion within the adrenal cortex; however PTH action has been shown to not be affected by the differential expression of LRP5 [232,233,234]. Instead, PTH and its receptor interact with the highly homologous LRP6 to activate β-catenin signaling independently of Wnt [235].

For the AC data set, only five genes were differentially expressed (*Gh1*, *Prl*, *C3*, *Ltbp2*, and *A4galt*), each of which was up-regulated in the PNS cows relative to the Control cows. Of these genes, *Gh1* and *Prl* were included in all the DEGs enriched in GO and KEGG terms, with the exception of *Gh1* not being included in the Extracellular Region term. Among the enriched terms, five GO and two KEGG terms were related to different portions of signal transduction, from receptor binding (Hormone Activity, Neuroactive Ligand–Receptor Interaction), to signal cascade (JAK-STAT signaling pathway, Positive Regulation of JAK-STAT Cascade), to the regulation of cellular functions (Positive Regulation of Gene Expression, Negative Regulation of Gene Expression, Negative Regulation of Apoptotic Process). Growth hormone 1 (GH1), encoded by *Gh1*, and prolactin (PRL), encoded by *Prl*, are both classically synthesized and released from the somatotrophs and lactotrophs of the anterior pituitary, respectively. Through the activation of JAK2 in the JAK-STAT signaling pathway [236,237], endocrine GH1 and PRL have been shown to promote adrenocortical cell hypertrophy, as well as enhance ACTH action in inducing androgen and glucocorticoid secretion [238,239,240,241,242]. However, there has been increasing evidence supporting the expression of these two genes within the periphery, particularly during embryonic and fetal development prior to pituitary ontogeny. The extra-pituitary expression of these genes postnatally has also been demonstrated, suggesting their persistent importance in mediating cellular function through autocrine and/or paracrine action, likely to complement their endocrine action [243,244,245]. Currently, there is a gap in the literature regarding whether these two hormones have extra-pituitary expression within the adrenal gland, though, based on their local actions in other tissues, it is probable they also exhibit similar autocrine/paracrine roles to enhance steroidogenesis and hypertrophy in the adrenocortical cells. However, it is not clear whether these two up-regulated genes were able to elicit a local cellular response, since there was no differential expression of glucocorticoids, mineralocorticoids, or androgens within the adrenal cortex of the PNS cows relative to the Control cows. There are two potential explanations for this phenomenon: (1) neither *Gh1* nor *Prl* were translated into functional proteins, since, though they were transcribed, it was not guaranteed that they would also be translated; or (2) assuming they were translated into proteins, the production of GH1 and PRL was not great enough to elicit a drastic difference in steroidogenesis in our mature cows exposed to prenatal stress. Overall, according to the DMGs and DEGs involved in the enriched signal transduction GO and KEGG terms, cell turnover within the adrenal cortex of the PNS cows may have been increased relative to the Control cows due to the promotion of apoptotic mechanisms. Also, steroidogenesis and hypertrophy may be enhanced upon ACTH stimulation of adrenocortical cells.

## 5. Conclusions

The results of this genome-wide assessment of DNAm and gene expression in the stress axis tissues of mature Brahman cows support our hypothesis that prenatal stress alters the postnatal methylome and transcriptome. Future studies focused on the change in methylomic and genomic dynamics within the neural and endocrine tissues of the same animal as it ages will increase the understanding of how various physiological processes, such as metabolism, stress response, and immune function, may adapt over time in individuals exposed to prenatal stress. However, the present impossibility of collecting these tissues more than once from the same animal precludes longitudinal studies, preventing the direct comparison of DNAm and gene expression postnatally and after maturity in the same animal.

The detection of differential methylation of the DNA and gene expression in the stress axis tissues indicated a potential impairment of the developmental and cellular processes in the prenatally stressed offspring. For example, the offspring may have had an increased health risk due to altered pituitary function, as well as adrenal-related disease and insufficiency. The induction of compensatory responses, such as the up-regulation of genes to ensure sufficient oxygenation and nutrient delivery to the neuronal tissue, reflects a possible beneficial effect of prenatal stress. It is of interest to note that there are at least five hormone-producing cell types within the anterior pituitary, and out of these cell types, corticotrophs only constitute about 20% [246]. Thus, there are likely other cell types within our PIT tissue samples that may have overshadowed the stress response effects experienced by our mature cattle from their prenatal transportation. Although bulk sequencing enables an overarching examination of the effect of prenatal transportation stress within the pituitary, going forward, a cell-specific analysis using single-cell sequencing may further clarify the effects of prenatal stress on the methylome and transcriptome of the tissues of the HPA axis.

There were several commonalities between the sample tissues in the type of terms that were enriched, suggesting that the consequences of prenatal stress on the methylome and transcriptome of cattle share similarities between the tissues of the stress axis, though further investigation is needed. There were also similarities in the type of terms enriched between the methylome and transcriptome within the sample tissues, yet none of the genes were both differentially methylated and expressed in any of the three sample tissues. Thus, potential differential gene expression caused by DNAm is unable to be confirmed with the transcriptomic data from the same tissue samples. A simultaneous proteomic analysis of the same tissue samples would be useful for determining whether there is a continued disparity from the methylated DNA to the transcribed RNA to the translated protein. This study contributes gene-level evidence regarding the postnatal effects of prenatal stress. Follow-up research is needed to fully understand the mechanisms behind prenatal stress and its potential long-term physiological or pathophysiological implications.

## Figures and Tables

**Table 1:** Number of differentially methylated (FDR < 0.05 and FDR < 0.15) CpG sites within promoter and gene body regions of genes in stress axis tissues. Sites were hypermethylated (hypomethylated) if stressed group had more (less) methylation than Control group.

Tissue	Hypermethylated		Hypomethylated		Unchanged	
	<0.05	<0.15	<0.05	<0.15	<0.05	<0.15
Paraventricular Nucleus	2	2	3	3	250,978	250,978
Anterior Pituitary	50	93	23	64	945,563	945,479
Adrenal Cortex	49	90	54	99	372,397	372,311

**Table 2:** Number of differentially expressed genes (FDR < 0.05 and FDR < 0.15) in stress axis tissues. Genes were up-regulated (down-regulated) if stressed group had increased (decreased) gene expression relative to Control group.

Tissue	Up-Regulated		Down-Regulated		Unchanged	
	<0.05	<0.15	<0.05	<0.15	<0.05	<0.15
Paraventricular Nucleus	1	6	0	0	13,268	13,263
Anterior Pituitary	4	25	5	24	12,217	12,177
Adrenal Cortex	4	5	0	0	11,104	11,103

**Table 3:** Breakdown of number of differentially methylated CpG sites (FDR < 0.05 and FDR < 0.15) located in either promoter regions or gene body regions (exons or introns) of each stress axis tissue. Sites are hypermethylated (hypomethylated) if stressed group has more (less) methylation than Control group.

Tissue		Hypermethylated			Hypomethylated		
		Exon	Intron	Promoter	Exon	Intron	Promoter
Paraventricular Nucleus	<0.05	1	1	0	0	1	1
	<0.15	1	1	0	0	1	1
Anterior Pituitary	<0.05	6	27	6	2	10	0
	<0.15	12	49	13	3	29	1
Adrenal Cortex	<0.05	4	25	6	2	27	4
	<0.15	11	45	15	13	46	13

**Table 4:** Functional annotation clusters grouping similar, redundant, and heterogenous annotation contents of the differentially methylated genes within the adrenal cortex of the stress axis tissues.

Adrenal Cortex	Functional Category	Term	Genes Involved	*p*-Value
Annotation Cluster 1	GO: Cellular Component	Presynaptic Membrane	*Grik4*, *Grin2a*	0.009
	GO: Cellular Component	Postsynaptic Membrane	*Grik4*, *Grin2a*	0.076
	GO: Molecular Function	Ligand-Gated Ion Channel Activity	*Grik4*, *Grin2a*	0.005
	GO: Molecular Function	Signaling Receptor Activity	*Grik4*, *Grin2a*	0.096
Annotation Cluster 2	KEGG Pathway	PI3K-Akt Signaling Pathway	*Degs2*, *Bcl2*, *Ppp2r3b*, *Ppp2r2c*	0.016
	KEGG Pathway	Sphingolipid Signaling Pathway	*Kcnq1*, *Bcl2*, *Ppp2r3b*, *Ppp2r2c*	0.028
	KEGG Pathway	Adrenergic Signaling in Cardiomyocytes	*Angpt2*, *Tnxb*, *Bcl2*, *Ppp2r3b*, *Ppp2r2c*	0.086
Annotation Cluster 3	GO: Cellular Component	Neuron Projection	*Ptprf*, *Grin2a*, *Kcnq1*	0.084
	GO: Cellular Component	Cell Surface	*Hbegf*, *Grin2a*, *Kcnq1*	0.223
	GO: Cellular Component	Endoplasmic Reticulum	*Grin2a*, *Kcnq1*	0.805

**Table 5:** Functional annotation clusters grouping similar, redundant, and heterogenous annotation contents from the differentially methylated genes within the anterior pituitary of the stress axis tissues.

Anterior Pituitary	Functional Category	Term	Genes Involved
Annotation Cluster 1	KEGG Pathway	Chemokine Signaling Pathway	*Pik3r6, Rac1, Gnb1*
	KEGG Pathway	PI3K-Akt Signaling Pathway	*Pik3r6, Rac1, Egfr, Gnb1*
	KEGG Pathway	Ras Signaling Pathway	*Rac1, Egfr, Gnb1*
	KEGG Pathway	Pathways in Cancer	*Rac1, Egfr, Gnb1, Rara, Rarb, Traf3*
	KEGG Pathway	Kaposi Sarcoma-Associated Herpesvirus Infection	*Pik3r6, Rac1, Gnb1, Traf3*
	KEGG Pathway	Human Cytomegalovirus Infection	*Rac1, Egfr, Gnb1*
Annotation Cluster 2	KEGG Pathway	MAPK Signaling Pathway	*Rac1, Egfr, Flna*
	KEGG Pathway	Focal Adhesion	*Rac1, Egfr, Flna*
	KEGG Pathway	Proteoglycans in Cancer	*Rac1, Egfr, Flna*

## Data Availability

The original contributions presented in this study are included in the article/Supplementary Material. Further inquiries can be directed to the corresponding author.

## Supplementary Materials

The supporting supplemental information can be downloaded at: https://www.mdpi.com/article/10.3390/genes16020191/s1. Supplementary Table S1. Locus and associated gene of differentially methylated (FDR < 0.15) CpG sites within promoter and gene body regions for each tissue of the stress axis. Supplementary Table S2. Ensembl ID of genes that were differentially expressed (FDR < 0.15) in stress axis tissues. Supplementary Table S3. Gene ontology and KEGG pathway functional annotation results for differentially expressed genes within the paraventricular nucleus of the stress axis tissues. Supplementary Table S4. Gene ontology and KEGG pathway functional annotation results for differentially methylated genes and differentially expressed genes within the anterior pituitary of the stress axis tissues. Supplementary Table S5. Gene ontology and KEGG pathway functional annotation results for differentially methylated genes and differentially expressed genes within the adrenal cortex of the stress axis tissues.
