# Supplementary material for: Evaluation of Prenatal Transportation Stress on DNA Methylation (DNAm) and Gene Expression in the Hypothalamic–Pituitary–Adrenal (HPA) Axis Tissues of Mature Brahman Cows
Source: Genes (Basel). 2025 Feb 4;16(2):191. doi: 10.3390/genes16020191 (PMC11855312; doi:10.3390/genes16020191)
Supplement: Supplementary file 1 [file genes-16-00191-s001.zip › genes-3410766-supplementary.pdf]

**Supplementary Table S1.** Locus and associated gene of differentially methylated (FDR < 0.15) CpG sites within promoter and gene body regions for each tissue of the stress axis. Positive (negative) logFC indicates that the gene was hypermethylated (hypomethylated), where methylation was greater (reduced) in the stressed group than control group.

| Tissue | Chromosome | Locus | Ensembl ID | Gene | Region | logFC | FDR |
|---|---|---|---|---|---|---|---|
| Paraventricular nucleus | 29 | 44386667 | ENSBTAG00000017681 | *Rin1* | Intron | -6.470 | < 0.001 |
| Paraventricular nucleus | 11 | 27359341 | ENSBTAG00000049988 | *Novel* | Promoter | -3.956 | 0.004 |
| Paraventricular nucleus | 7 | 38952896 | ENSBTAG00000011398 | *Prr7* | Exon | 4.831 | 0.031 |
| Paraventricular nucleus | 29 | 46187829 | ENSBTAG00000021999 | *Cpt1a* | Intron | 4.485 | 0.004 |
| Anterior pituitary | 11 | 93248767 | ENSBTAG00000006716 | *Ptgs1* | Exon | -5.046 | < 0.001 |
| Anterior pituitary | 17 | 71213263 | ENSBTAG00000053568 | *Znf70* | Exon | -3.704 | 0.021 |
| Anterior pituitary | 29 | 46292201 | ENSBTAG00000022185 | *Ighmbp2* | Exon | -2.937 | 0.084 |
| Anterior pituitary | 14 | 3652718 | ENSBTAG00000047488 | *Kcnk9* | Intron | -5.630 | < 0.001 |
| Anterior pituitary | 24 | 57483548 | ENSBTAG00000005412 | *Nedd4l* | Intron | -5.989 | 0.005 |
| Anterior pituitary | 23 | 49631975 | ENSBTAG00000016397 | *Cdyl* | Intron | -4.114 | 0.006 |
| Anterior pituitary | 14 | 7212356 | ENSBTAG00000017731 | *Zfat* | Intron | -4.118 | 0.013 |
| Anterior pituitary | 8 | 8938190 | ENSBTAG00000021632 | *Msra* | Intron | -4.203 | 0.017 |
| Anterior pituitary | 25 | 38293736 | ENSBTAG00000053390 | *Rac1* | Intron | -4.838 | 0.017 |
| Anterior pituitary | 29 | 42882704 | ENSBTAG00000000244 | *Nrxn2* | Intron | -4.056 | 0.030 |
| Anterior pituitary | 3 | 120221833 | ENSBTAG00000005168 | *Crocc2* | Intron | -5.416 | 0.030 |
| Anterior pituitary | 23 | 5065713 | ENSBTAG00000012973 | *Gfral* | Intron | -5.113 | 0.040 |
| Anterior pituitary | 11 | 80696602 | ENSBTAG00000051528 | *Novel* | Intron | -3.080 | 0.047 |
| Anterior pituitary | 23 | 43402732 | ENSBTAG00000012039 | *Phactr1* | Intron | -3.568 | 0.053 |

| | | | | | | | |
|---|---|---|---|---|---|---|---|
| Anterior pituitary | 5 | 116785368 | ENSBTAG00000008036 | *Celsr1* | Intron | -2.393 | 0.055 |
| Anterior pituitary | 25 | 1154013 | ENSBTAG00000015889 | *Clcn7* | Intron | -3.081 | 0.055 |
| Anterior pituitary | 7 | 44224571 | ENSBTAG00000045762 | *Scamp4* | Intron | -4.064 | 0.068 |
| Anterior pituitary | 5 | 116677042 | ENSBTAG00000008036 | *Celsr1* | Intron | -2.607 | 0.091 |
| Anterior pituitary | 16 | 50907277 | ENSBTAG00000000215 | *Gnb1* | Intron | -4.569 | 0.094 |
| Anterior pituitary | 9 | 97903579 | ENSBTAG00000018996 | *Prkn* | Intron | -4.672 | 0.102 |
| Anterior pituitary | 2 | 133332481 | ENSBTAG00000004554 | *Capzb* | Intron | -3.772 | 0.103 |
| Anterior pituitary | 8 | 108822975 | ENSBTAG00000015936 | *Brinp1* | Intron | -3.867 | 0.104 |
| Anterior pituitary | 5 | 117219851 | ENSBTAG00000012291 | *Tbc1d22a* | Intron | -2.741 | 0.108 |
| Anterior pituitary | 24 | 2579653 | ENSBTAG00000004650 | *Znf516* | Intron | -4.645 | 0.120 |
| Anterior pituitary | 17 | 40307146 | ENSBTAG00000016679 | *Etfdh* | Intron | -3.204 | 0.124 |
| Anterior pituitary | 13 | 69983385 | ENSBTAG00000009165 | *Lpin3* | Intron | -3.089 | 0.133 |
| Anterior pituitary | 17 | 71976195 | ENSBTAG00000006938 | *Ppm1f* | Intron | -2.854 | 0.140 |
| Anterior pituitary | 2 | 133332482 | ENSBTAG00000004554 | *Capzb* | Intron | -3.989 | 0.140 |
| Anterior pituitary | 14 | 15102278 | ENSBTAG00000010275 | *Washc5* | Intron | -3.005 | 0.144 |
| Anterior pituitary | 11 | 105365461 | ENSBTAG00000047998 | *Col5a1* | Intron | -3.337 | 0.144 |
| Anterior pituitary | 24 | 57360738 | ENSBTAG00000005412 | *Nedd4l* | Intron | -3.199 | 0.145 |
| Anterior pituitary | 21 | 64223795 | ENSBTAG00000018019 | *Bcl11b* | Intron | -2.468 | 0.147 |
| Anterior pituitary | 16 | 50907277 | ENSBTAG00000000215 | *Gnb1* | Promoter | -4.569 | 0.094 |
| Anterior pituitary | 1 | 145938819 | ENSBTAG00000003148 | *Mcm3ap* | Exon | 4.004 | 0.003 |
| Anterior pituitary | 10 | 88237994 | ENSBTAG00000025329 | *Irf2bpl* | Exon | 5.001 | 0.003 |

| | | | | | | | |
|---|---|---|---|---|---|---|---|
| Anterior pituitary | 2 | 47228801 | ENSBTAG00000018125 | *Kif5c* | Exon | 4.703 | 0.004 |
| Anterior pituitary | X | 36972236 | ENSBTAG00000020551 | *Abcd1* | Exon | 4.293 | 0.005 |
| Anterior pituitary | 29 | 36040470 | ENSBTAG00000017602 | *Tmem45b* | Exon | 2.359 | 0.026 |
| Anterior pituitary | 1 | 145938818 | ENSBTAG00000003148 | *Mcm3ap* | Exon | 3.884 | 0.030 |
| Anterior pituitary | 17 | 53175178 | ENSBTAG00000004197 | *B3gnt4* | Exon | 4.225 | 0.059 |
| Anterior pituitary | 21 | 67572831 | ENSBTAG00000013475 | *Traf3* | Exon | 4.744 | 0.079 |
| Anterior pituitary | 19 | 28320462 | ENSBTAG00000016344 | *Pik3r6* | Exon | 3.189 | 0.108 |
| Anterior pituitary | 12 | 36114772 | ENSBTAG00000020400 | *Gja3* | Exon | 3.376 | 0.124 |
| Anterior pituitary | 26 | 51984498 | ENSBTAG00000019519 | *Pwwp2b* | Exon | 3.899 | 0.140 |
| Anterior pituitary | 12 | 36114870 | ENSBTAG00000020400 | *Gja3* | Exon | 3.124 | 0.144 |
| Anterior pituitary | 25 | 41133297 | ENSBTAG00000002474 | *Mad1l1* | Intron | 5.487 | < 0.001 |
| Anterior pituitary | 21 | 65109237 | ENSBTAG00000009294 | *Degs2* | Intron | 5.949 | < 0.001 |
| Anterior pituitary | 28 | 28381710 | ENSBTAG00000013651 | *Dnajb12* | Intron | 5.705 | < 0.001 |
| Anterior pituitary | 21 | 67582532 | ENSBTAG00000013475 | *Traf3* | Intron | 5.896 | < 0.001 |
| Anterior pituitary | 21 | 65334374 | ENSBTAG00000007233 | *Wdr25* | Intron | 5.693 | < 0.001 |
| Anterior pituitary | 1 | 157344691 | ENSBTAG00000000746 | *Kat2b* | Intron | 4.049 | < 0.001 |
| Anterior pituitary | 21 | 67582366 | ENSBTAG00000013475 | *Traf3* | Intron | 6.914 | < 0.001 |
| Anterior pituitary | 21 | 65560785 | ENSBTAG00000052188 | *Novel* | Intron | 5.037 | < 0.001 |
| Anterior pituitary | 13 | 45113447 | ENSBTAG00000002683 | *Pfkp* | Intron | 4.028 | < 0.001 |
| Anterior pituitary | 13 | 45113470 | ENSBTAG00000002683 | *Pfkp* | Intron | 4.425 | < 0.001 |
| Anterior pituitary | 25 | 41133363 | ENSBTAG00000002474 | *Mad1l1* | Intron | 4.100 | < 0.001 |

| | | | | | | | |
|---|---|---|---|---|---|---|---|
| Anterior pituitary | 28 | 26726528 | ENSBTAG00000008583 | *Pald1* | Intron | 5.479 | 0.002 |
| Anterior pituitary | 21 | 67042201 | ENSBTAG00000007007 | *Wdr20* | Intron | 5.687 | 0.003 |
| Anterior pituitary | 21 | 65519842 | ENSBTAG00000052188 | *Novel* | Intron | 4.987 | 0.003 |
| Anterior pituitary | 27 | 13938110 | ENSBTAG00000049918 | *Novel* | Intron | 5.316 | 0.004 |
| Anterior pituitary | 21 | 67675017 | ENSBTAG00000016456 | *Cdc42bpb* | Intron | 6.060 | 0.004 |
| Anterior pituitary | 21 | 32852610 | ENSBTAG00000052746 | *Novel* | Intron | 5.035 | 0.007 |
| Anterior pituitary | 4 | 113664639 | ENSBTAG00000048379 | *Novel* | Intron | 4.278 | 0.007 |
| Anterior pituitary | 2 | 62056503 | ENSBTAG00000026842 | *Zranb3* | Intron | 5.215 | 0.018 |
| Anterior pituitary | 12 | 35947852 | ENSBTAG00000011726 | *Cryl1* | Intron | 3.136 | 0.024 |
| Anterior pituitary | 9 | 9946287 | ENSBTAG00000038190 | *Smap1* | Intron | 3.477 | 0.026 |
| Anterior pituitary | 29 | 36040470 | ENSBTAG00000017602 | *Tmem45b* | Intron | 2.359 | 0.026 |
| Anterior pituitary | 22 | 978475 | ENSBTAG00000011628 | *Egfr* | Intron | 5.018 | 0.029 |
| Anterior pituitary | 25 | 41440842 | ENSBTAG00000000642 | *Ints1* | Intron | 4.939 | 0.040 |
| Anterior pituitary | 21 | 67124466 | ENSBTAG00000010993 | *Znf839* | Intron | 5.417 | 0.047 |
| Anterior pituitary | 22 | 60459260 | ENSBTAG00000008778 | *Chst13* | Intron | 4.011 | 0.047 |
| Anterior pituitary | 17 | 71217104 | ENSBTAG00000053568 | *Znf70* | Intron | 3.876 | 0.049 |
| Anterior pituitary | 29 | 49614847 | ENSBTAG00000010447 | *Lsp1* | Intron | 4.815 | 0.055 |
| Anterior pituitary | 13 | 81050104 | ENSBTAG00000007917 | *Tshz2* | Intron | 4.689 | 0.055 |
| Anterior pituitary | 21 | 65575613 | ENSBTAG00000052188 | *Novel* | Intron | 3.782 | 0.055 |
| Anterior pituitary | 21 | 30363839 | ENSBTAG00000001396 | *Adamts7* | Intron | 2.840 | 0.055 |
| Anterior pituitary | 21 | 64766026 | ENSBTAG00000013491 | *Eml1* | Intron | 3.857 | 0.057 |

| | | | | | | | |
|---|---|---|---|---|---|---|---|
| Anterior pituitary | 28 | 30872541 | ENSBTAG00000023039 | *Dusp13* | Intron | 4.489 | 0.059 |
| Anterior pituitary | 19 | 40589680 | ENSBTAG00000012500 | *Rara* | Intron | 4.504 | 0.066 |
| Anterior pituitary | 21 | 67187837 | ENSBTAG00000012143 | *Tecpr2* | Intron | 4.945 | 0.070 |
| Anterior pituitary | 15 | 33391984 | ENSBTAG00000000842 | *Ubash3b* | Intron | 4.018 | 0.084 |
| Anterior pituitary | 5 | 63035166 | ENSBTAG00000030994 | *Novel* | Intron | 2.985 | 0.085 |
| Anterior pituitary | 7 | 4453329 | ENSBTAG00000010379 | *Crtc1* | Intron | 3.746 | 0.095 |
| Anterior pituitary | 2 | 127345528 | ENSBTAG00000003069 | *Man1c1* | Intron | 3.401 | 0.103 |
| Anterior pituitary | 6 | 111262565 | ENSBTAG00000013736 | *Prom1* | Intron | 3.330 | 0.103 |
| Anterior pituitary | 17 | 52674357 | ENSBTAG00000021595 | *Hip1r* | Intron | 3.091 | 0.103 |
| Anterior pituitary | 4 | 103150462 | ENSBTAG00000017860 | *Hipk2* | Intron | 4.728 | 0.108 |
| Anterior pituitary | 29 | 49613364 | ENSBTAG00000010447 | *Lsp1* | Intron | 4.522 | 0.108 |
| Anterior pituitary | 12 | 35947801 | ENSBTAG00000011726 | *Cryl1* | Intron | 2.784 | 0.116 |
| Anterior pituitary | 1 | 145948497 | ENSBTAG00000003148 | *Mcm3ap* | Intron | 4.043 | 0.120 |
| Anterior pituitary | 25 | 37679539 | ENSBTAG00000019181 | *Baiap2l1* | Intron | 4.513 | 0.124 |
| Anterior pituitary | 18 | 11606120 | ENSBTAG00000009918 | *Gse1* | Intron | 4.658 | 0.130 |
| Anterior pituitary | 14 | 1816781 | ENSBTAG00000006385 | *Adgrb1* | Intron | 4.246 | 0.140 |
| Anterior pituitary | X | 37404529 | ENSBTAG00000011190 | *Flna* | Intron | 4.318 | 0.144 |
| Anterior pituitary | 7 | 39214893 | ENSBTAG00000006368 | *N4bp3* | Promoter | 6.093 | < 0.001 |
| Anterior pituitary | 27 | 40383054 | ENSBTAG00000011518 | *Rarb* | Promoter | 5.650 | < 0.001 |
| Anterior pituitary | 29 | 36040470 | ENSBTAG00000017602 | *Tmem45b* | Promoter | 2.359 | 0.026 |
| Anterior pituitary | 22 | 978475 | ENSBTAG00000011628 | *Egfr* | Promoter | 5.018 | 0.029 |

| | | | | | | | |
|---|---|---|---|---|---|---|---|
| Anterior pituitary | 22 | 60459260 | ENSBTAG00000008778 | *Chst13* | Promoter | 4.011 | 0.047 |
| Anterior pituitary | 17 | 53175558 | ENSBTAG00000004197 | *B3gnt4* | Promoter | 3.878 | 0.047 |
| Anterior pituitary | 29 | 49614847 | ENSBTAG00000010447 | *Lsp1* | Promoter | 4.815 | 0.055 |
| Anterior pituitary | 28 | 30872541 | ENSBTAG00000023039 | *Dusp13* | Promoter | 4.489 | 0.059 |
| Anterior pituitary | 17 | 53175178 | ENSBTAG00000004197 | *B3gnt4* | Promoter | 4.225 | 0.059 |
| Anterior pituitary | 21 | 67572831 | ENSBTAG00000013475 | *Traf3* | Promoter | 4.744 | 0.079 |
| Anterior pituitary | 29 | 49613364 | ENSBTAG00000010447 | *Lsp1* | Promoter | 4.522 | 0.108 |
| Anterior pituitary | 12 | 36114772 | ENSBTAG00000020400 | *Gja3* | Promoter | 3.376 | 0.124 |
| Anterior pituitary | 12 | 36114870 | ENSBTAG00000020400 | *Gja3* | Promoter | 3.124 | 0.144 |
| Adrenal cortex | 23 | 15598218 | ENSBTAG00000038916 | *Prickle4* | Exon | -5.164 | < 0.001 |
| Adrenal cortex | 1 | 97612885 | ENSBTAG00000043157 | *Telomerase-vert* | Exon | -4.856 | 0.026 |
| Adrenal cortex | 18 | 1833670 | ENSBTAG00000004766 | *Clec18c* | Exon | -3.937 | 0.051 |
| Adrenal cortex | 1 | 97612925 | ENSBTAG00000043157 | *Telomerase-vert* | Exon | -4.676 | 0.055 |
| Adrenal cortex | 22 | 14817168 | ENSBTAG00000006563 | *Klhl40* | Exon | -2.987 | 0.066 |
| Adrenal cortex | 7 | 51544252 | ENSBTAG00000021766 | *Hbegf* | Exon | -4.489 | 0.084 |
| Adrenal cortex | 1 | 97612863 | ENSBTAG00000043157 | *Telomerase-vert* | Exon | -4.539 | 0.094 |
| Adrenal cortex | 1 | 97612975 | ENSBTAG00000043157 | *Telomerase-vert* | Exon | -4.511 | 0.099 |
| Adrenal cortex | 1 | 97612881 | ENSBTAG00000043157 | *Telomerase-vert* | Exon | -4.474 | 0.110 |
| Adrenal cortex | 6 | 117225713 | ENSBTAG00000008480 | *Ctbp1* | Exon | -2.788 | 0.115 |
| Adrenal cortex | 15 | 39680738 | ENSBTAG00000011914 | *Rassf10* | Exon | -3.386 | 0.134 |
| Adrenal cortex | 23 | 33335649 | ENSBTAG00000009798 | *Dcdc2* | Exon | -4.366 | 0.138 |

| | | | | | | | |
|---|---|---|---|---|---|---|---|
| Adrenal cortex | 23 | 33335649 | ENSBTAG00000049612 | *Novel* | Exon | -4.366 | 0.138 |
| Adrenal cortex | 23 | 15598218 | ENSBTAG00000009861 | *Frs3* | Intron | -5.164 | < 0.001 |
| Adrenal cortex | 5 | 116677042 | ENSBTAG00000008036 | *Celsr1* | Intron | -3.823 | < 0.001 |
| Adrenal cortex | 11 | 19447249 | ENSBTAG00000014310 | *Heatr5b* | Intron | -4.072 | < 0.001 |
| Adrenal cortex | 5 | 70888822 | ENSBTAG00000027064 | *Btbd11* | Intron | -3.758 | < 0.001 |
| Adrenal cortex | 29 | 45866064 | ENSBTAG00000005903 | *Lrp5* | Intron | -5.756 | < 0.001 |
| Adrenal cortex | 25 | 39176867 | ENSBTAG00000030690 | *Radil* | Intron | -3.271 | < 0.001 |
| Adrenal cortex | 29 | 44386667 | ENSBTAG00000017681 | *Rin1* | Intron | -5.650 | < 0.001 |
| Adrenal cortex | 25 | 8619161 | ENSBTAG00000007887 | *Grin2a* | Intron | -3.863 | < 0.001 |
| Adrenal cortex | 12 | 21358246 | ENSBTAG00000010353 | *Atp7b* | Intron | -3.300 | 0.002 |
| Adrenal cortex | 3 | 119582487 | ENSBTAG00000054242 | *Akap17a* | Intron | -3.435 | 0.004 |
| Adrenal cortex | 29 | 45864419 | ENSBTAG00000005903 | *Lrp5* | Intron | -5.224 | 0.004 |
| Adrenal cortex | 3 | 102424601 | ENSBTAG00000000253 | *Ptprf* | Intron | -3.175 | 0.005 |
| Adrenal cortex | 28 | 26916830 | ENSBTAG00000021177 | *Adamts14* | Intron | -3.904 | 0.005 |
| Adrenal cortex | 29 | 48101424 | ENSBTAG00000003171 | *Shank2* | Intron | -3.094 | 0.008 |
| Adrenal cortex | 5 | 116676997 | ENSBTAG00000008036 | *Celsr1* | Intron | -3.055 | 0.008 |
| Adrenal cortex | 25 | 14519765 | ENSBTAG00000015191 | *Abcc6* | Intron | -3.601 | 0.008 |
| Adrenal cortex | 29 | 44390533 | ENSBTAG00000017689 | *Brms1* | Intron | -5.057 | 0.012 |
| Adrenal cortex | 24 | 61482371 | ENSBTAG00000019302 | *Bcl2* | Intron | -3.336 | 0.012 |
| Adrenal cortex | 1 | 107397483 | ENSBTAG00000052570 | *Novel* | Intron | -2.790 | 0.017 |
| Adrenal cortex | 29 | 44878179 | ENSBTAG00000019700 | *Pc* | Intron | -4.948 | 0.023 |

| | | | | | | | |
|---|---|---|---|---|---|---|---|
| Adrenal cortex | 7 | 18343766 | ENSBTAG00000017661 | *Rfx2* | Intron | -3.217 | 0.028 |
| Adrenal cortex | 24 | 2309686 | ENSBTAG00000022890 | *Mbp* | Intron | -3.132 | 0.031 |
| Adrenal cortex | 7 | 94337794 | ENSBTAG00000054976 | *Mctp1* | Intron | -2.945 | 0.037 |
| Adrenal cortex | 23 | 48068513 | ENSBTAG00000005980 | *Rreb1* | Intron | -2.946 | 0.038 |
| Adrenal cortex | 18 | 1833692 | ENSBTAG00000004766 | *Clec18c* | Intron | -4.682 | 0.046 |
| Adrenal cortex | 5 | 118386023 | ENSBTAG00000022986 | *Tafa5* | Intron | -2.726 | 0.048 |
| Adrenal cortex | 9 | 95149463 | ENSBTAG00000010334 | *Sytl3* | Intron | -2.877 | 0.050 |
| Adrenal cortex | 22 | 56147911 | ENSBTAG00000001814 | *Plxnd1* | Intron | -2.733 | 0.065 |
| Adrenal cortex | 29 | 29782354 | ENSBTAG00000006804 | *Kirrel3* | Intron | -4.304 | 0.085 |
| Adrenal cortex | 23 | 27282007 | ENSBTAG00000001444 | *Tnxb* | Intron | -3.446 | 0.089 |
| Adrenal cortex | 1 | 88381447 | ENSBTAG00000052803 | *Novel* | Intron | -3.354 | 0.091 |
| Adrenal cortex | 23 | 47508874 | ENSBTAG00000019234 | *Bmp6* | Intron | -2.476 | 0.093 |
| Adrenal cortex | 11 | 2882770 | ENSBTAG00000006019 | *Novel* | Intron | -4.283 | 0.098 |
| Adrenal cortex | 6 | 102859854 | ENSBTAG00000020598 | *Ppp2r2c* | Intron | -3.376 | 0.100 |
| Adrenal cortex | 19 | 18561598 | ENSBTAG00000016972 | *Rar11fip4* | Intron | -2.777 | 0.110 |
| Adrenal cortex | 6 | 68550190 | ENSBTAG00000020648 | *Scfd2* | Intron | -4.083 | 0.111 |
| Adrenal cortex | 24 | 61421869 | ENSBTAG00000019302 | *Bcl2* | Intron | -3.517 | 0.112 |
| Adrenal cortex | 3 | 102365363 | ENSBTAG00000002078 | *Kdm4a* | Intron | -2.340 | 0.115 |
| Adrenal cortex | 2 | 88043563 | ENSBTAG00000016334 | *Satb2* | Intron | -4.366 | 0.125 |
| Adrenal cortex | 25 | 39022708 | ENSBTAG00000012049 | *Wipi2* | Intron | -2.838 | 0.125 |
| Adrenal cortex | 19 | 22347808 | ENSBTAG00000039819 | *Rph3al* | Intron | -3.458 | 0.130 |

| | | | | | | | |
|---|---|---|---|---|---|---|---|
| Adrenal cortex | 5 | 118386054 | ENSBTAG00000022986 | *Tafa5* | Intron | -2.492 | 0.133 |
| Adrenal cortex | 9 | 9547961 | ENSBTAG00000002171 | *Fam135a* | Intron | -2.675 | 0.137 |
| Adrenal cortex | 25 | 29140176 | ENSBTAG00000008718 | *Galnt17* | Intron | -3.999 | 0.138 |
| Adrenal cortex | 13 | 46202219 | ENSBTAG00000014194 | *Adarb2* | Intron | -3.694 | 0.140 |
| Adrenal cortex | 25 | 41040241 | ENSBTAG00000002474 | *Mad1l1* | Intron | -2.633 | 0.143 |
| Adrenal cortex | 29 | 44390533 | ENSBTAG00000017681 | *Rin1* | Promoter | -5.057 | 0.012 |
| Adrenal cortex | 1 | 97612885 | ENSBTAG00000043157 | *Telomerase-vert* | Promoter | -4.856 | 0.026 |
| Adrenal cortex | 1 | 97612914 | ENSBTAG00000043157 | *Telomerase-vert* | Promoter | -4.846 | 0.027 |
| Adrenal cortex | 7 | 43956209 | ENSBTAG00000040334 | *Mex3d* | Promoter | -4.767 | 0.033 |
| Adrenal cortex | 1 | 97612925 | ENSBTAG00000043157 | *Telomerase-vert* | Promoter | -4.676 | 0.055 |
| Adrenal cortex | 28 | 28073364 | ENSBTAG00000011014 | *Chst3* | Promoter | -3.295 | 0.066 |
| Adrenal cortex | 22 | 14817168 | ENSBTAG00000006563 | *Klhl40* | Promoter | -2.987 | 0.066 |
| Adrenal cortex | 1 | 97612863 | ENSBTAG00000043157 | *Telomerase-vert* | Promoter | -4.539 | 0.094 |
| Adrenal cortex | 1 | 97612975 | ENSBTAG00000043157 | *Telomerase-vert* | Promoter | -4.511 | 0.099 |
| Adrenal cortex | 1 | 97612881 | ENSBTAG00000043157 | *Telomerase-vert* | Promoter | -4.474 | 0.110 |
| Adrenal cortex | 2 | 88043563 | ENSBTAG00000016334 | *Satb2* | Promoter | -4.366 | 0.125 |
| Adrenal cortex | 23 | 33335649 | ENSBTAG00000009798 | *Dcdc2* | Promoter | -4.366 | 0.138 |
| Adrenal cortex | 23 | 33335649 | ENSBTAG00000049612 | *Novel* | Promoter | -4.366 | 0.138 |
| Adrenal cortex | 29 | 36040470 | ENSBTAG00000017602 | *Tmem45b* | Exon | 4.255 | < 0.001 |
| Adrenal cortex | 17 | 28956529 | ENSBTAG00000017493 | *Jade1* | Exon | 4.304 | < 0.001 |
| Adrenal cortex | 17 | 53175178 | ENSBTAG00000004197 | *B3gnt4* | Exon | 3.647 | 0.003 |

| | | | | | | | |
|---|---|---|---|---|---|---|---|
| Adrenal cortex | 1 | 56659681 | ENSBTAG00000001209 | *Phldb2* | Exon | 3.967 | 0.004 |
| Adrenal cortex | 25 | 20589987 | ENSBTAG00000017391 | *Hs3st2* | Exon | 3.144 | 0.061 |
| Adrenal cortex | 19 | 44116404 | ENSBTAG00000001615 | *Rundc3a* | Exon | 3.601 | 0.062 |
| Adrenal cortex | 4 | 65648051 | ENSBTAG00000014381 | *Crhr2* | Exon | 4.240 | 0.084 |
| Adrenal cortex | 9 | 96309458 | ENSBTAG00000008540 | *Slc22a1* | Exon | 4.301 | 0.117 |
| Adrenal cortex | 27 | 5611840 | ENSBTAG00000011034 | *Angpt2* | Exon | 3.363 | 0.120 |
| Adrenal cortex | 23 | 49522799 | ENSBTAG00000054777 | *Ppp1r3g* | Exon | 3.556 | 0.124 |
| Adrenal cortex | 1 | 626142 | ENSBTAG00000054829 | *Novel* | Exon | 4.375 | 0.133 |
| Adrenal cortex | 21 | 67675017 | ENSBTAG00000016456 | *Cdc42bpb* | Intron | 6.596 | < 0.001 |
| Adrenal cortex | 29 | 36040470 | ENSBTAG00000017602 | *Tmem45b* | Intron | 4.255 | < 0.001 |
| Adrenal cortex | 21 | 65109237 | ENSBTAG00000009294 | *Degs2* | Intron | 5.319 | < 0.001 |
| Adrenal cortex | 17 | 71378945 | ENSBTAG00000024708 | *Cabin1* | Intron | 6.248 | < 0.001 |
| Adrenal cortex | 21 | 65519842 | ENSBTAG00000052188 | *Novel* | Intron | 4.152 | < 0.001 |
| Adrenal cortex | 3 | 112898597 | ENSBTAG00000020173 | *Inpp5d* | Intron | 3.757 | < 0.001 |
| Adrenal cortex | 14 | 7149442 | ENSBTAG00000017731 | *Zfat* | Intron | 4.594 | < 0.001 |
| Adrenal cortex | 7 | 19464815 | ENSBTAG00000021134 | *Dpp9* | Intron | 5.238 | < 0.001 |
| Adrenal cortex | 26 | 46408182 | ENSBTAG00000031890 | *Dock1* | Intron | 3.603 | 0.001 |
| Adrenal cortex | 14 | 431192 | ENSBTAG00000010276 | *Recql4* | Intron | 5.533 | 0.002 |
| Adrenal cortex | 28 | 30872541 | ENSBTAG00000023039 | *Dusp13* | Intron | 5.042 | 0.002 |
| Adrenal cortex | 25 | 40647131 | ENSBTAG00000026194 | *Amz1* | Intron | 3.665 | 0.002 |
| Adrenal cortex | 25 | 1196552 | ENSBTAG00000007245 | *Ift140* | Intron | 2.904 | 0.004 |

| | | | | | | | |
|---|---|---|---|---|---|---|---|
| Adrenal cortex | 16 | 80393271 | ENSBTAG00000004789 | *Lgr6* | Intron | 4.388 | 0.006 |
| Adrenal cortex | 29 | 48774600 | ENSBTAG00000010986 | *Kcnq1* | Intron | 3.163 | 0.013 |
| Adrenal cortex | 4 | 116420656 | ENSBTAG00000021941 | *Dpp6* | Intron | 3.241 | 0.015 |
| Adrenal cortex | 15 | 31586671 | ENSBTAG00000017146 | *Grik4* | Intron | 3.303 | 0.015 |
| Adrenal cortex | 29 | 24864411 | ENSBTAG00000018431 | *Nav2* | Intron | 3.241 | 0.018 |
| Adrenal cortex | 29 | 45864383 | ENSBTAG00000005903 | *Lrp5* | Intron | 3.785 | 0.023 |
| Adrenal cortex | 16 | 42887101 | ENSBTAG00000019818 | *Casz1* | Intron | 3.037 | 0.029 |
| Adrenal cortex | 5 | 106562815 | ENSBTAG00000049163 | *Tspan9* | Intron | 2.809 | 0.030 |
| Adrenal cortex | 25 | 41983323 | ENSBTAG00000046142 | *Prkar1b* | Intron | 2.992 | 0.031 |
| Adrenal cortex | 29 | 49613364 | ENSBTAG00000010447 | *Lsp1* | Intron | 4.626 | 0.031 |
| Adrenal cortex | 20 | 62175099 | ENSBTAG00000017222 | *Ctnnd2* | Intron | 3.251 | 0.036 |
| Adrenal cortex | 18 | 5580456 | ENSBTAG00000015894 | *Wwox* | Intron | 4.725 | 0.038 |
| Adrenal cortex | 12 | 35979955 | ENSBTAG00000011726 | *Cryl1* | Intron | 2.995 | 0.053 |
| Adrenal cortex | 22 | 56555762 | ENSBTAG00000004512 | *Mkrn2* | Intron | 3.023 | 0.055 |
| Adrenal cortex | 18 | 11570314 | ENSBTAG00000009918 | *Gse1* | Intron | 4.635 | 0.057 |
| Adrenal cortex | 17 | 72446028 | ENSBTAG00000018048 | *Med15* | Intron | 4.387 | 0.060 |
| Adrenal cortex | 29 | 49614903 | ENSBTAG00000010447 | *Lsp1* | Intron | 4.602 | 0.078 |
| Adrenal cortex | 10 | 83951221 | ENSBTAG00000014189 | *Rgs6* | Intron | 3.086 | 0.086 |
| Adrenal cortex | 12 | 15146898 | ENSBTAG00000009415 | *Nufip1* | Intron | 2.563 | 0.089 |
| Adrenal cortex | 13 | 18741613 | ENSBTAG00000014991 | *Pard3* | Intron | 3.398 | 0.091 |
| Adrenal cortex | 3 | 7931827 | ENSBTAG00000021842 | *Fcgr2b* | Intron | 3.282 | 0.093 |

| | | | | | | | |
|---|---|---|---|---|---|---|---|
| Adrenal cortex | 21 | 67234200 | ENSBTAG00000012143 | *Tecpr2* | Intron | 2.547 | 0.094 |
| Adrenal cortex | 6 | 117503633 | ENSBTAG00000050808 | *Cplx1* | Intron | 2.641 | 0.098 |
| Adrenal cortex | 25 | 39618049 | ENSBTAG00000004035 | *Sdk1* | Intron | 2.859 | 0.098 |
| Adrenal cortex | 25 | 14611540 | ENSBTAG00000017759 | *Novel* | Intron | 4.379 | 0.098 |
| Adrenal cortex | 4 | 114320224 | ENSBTAG00000002917 | *Prkag2* | Intron | 2.504 | 0.100 |
| Adrenal cortex | 17 | 71375783 | ENSBTAG00000024708 | *Cabin1* | Intron | 2.721 | 0.110 |
| Adrenal cortex | 19 | 33201956 | ENSBTAG00000025078 | *Lrrc75a* | Intron | 4.315 | 0.112 |
| Adrenal cortex | 27 | 5611840 | ENSBTAG00000011032 | *Mcph1* | Intron | 3.363 | 0.120 |
| Adrenal cortex | 6 | 114321275 | ENSBTAG00000015926 | *Ablim2* | Intron | 2.844 | 0.123 |
| Adrenal cortex | 25 | 41983322 | ENSBTAG00000046142 | *Prkar1b* | Intron | 2.554 | 0.134 |
| Adrenal cortex | 10 | 83951276 | ENSBTAG00000014189 | *Rgs6* | Intron | 3.152 | 0.143 |
| Adrenal cortex | 29 | 36040470 | ENSBTAG00000017602 | *Tmem45b* | Promoter | 4.255 | < 0.001 |
| Adrenal cortex | 28 | 30872541 | ENSBTAG00000023039 | *Dusp13* | Promoter | 5.042 | 0.002 |
| Adrenal cortex | 17 | 53175178 | ENSBTAG00000004197 | *B3gnt4* | Promoter | 3.647 | 0.003 |
| Adrenal cortex | 25 | 1196552 | ENSBTAG00000007246 | *Tmem204* | Promoter | 2.904 | 0.004 |
| Adrenal cortex | 21 | 33002648 | ENSBTAG00000050043 | *5s_rRNA* | Promoter | 3.326 | 0.006 |
| Adrenal cortex | 29 | 49613364 | ENSBTAG00000010447 | *Lsp1* | Promoter | 4.626 | 0.031 |
| Adrenal cortex | 19 | 44116404 | ENSBTAG00000001615 | *Rundc3a* | Promoter | 3.601 | 0.062 |
| Adrenal cortex | 29 | 49614903 | ENSBTAG00000010447 | *Lsp1* | Promoter | 4.602 | 0.078 |
| Adrenal cortex | 4 | 65648051 | ENSBTAG00000014381 | *Crhr2* | Promoter | 4.240 | 0.084 |
| Adrenal cortex | 21 | 33026011 | ENSBTAG00000055146 | *5s_rRNA* | Promoter | 2.528 | 0.099 |

| | | | | | | | |
|---|---|---|---|---|---|---|---|
| Adrenal cortex | 19 | 55694759 | ENSBTAG00000030175 | *Fbf1* | Promoter | 4.259 | 0.099 |
| Adrenal cortex | 4 | 114320224 | ENSBTAG00000002917 | *Prkag2* | Promoter | 2.504 | 0.100 |
| Adrenal cortex | 27 | 5611840 | ENSBTAG00000011034 | *Angpt2* | Promoter | 3.363 | 0.120 |
| Adrenal cortex | 23 | 49522799 | ENSBTAG00000054777 | *Ppp1r3g* | Promoter | 3.556 | 0.124 |
| Adrenal cortex | 1 | 626142 | ENSBTAG00000054829 | *Novel* | Promoter | 4.375 | 0.133 |

Table is organized by tissue (PVN, PIT, AC), then logFC (negative, positive), and then region (exon, intron, promoter).

**Supplementary Table S2.** Ensembl ID of genes that were differentially expressed (FDR < 0.15) in stress axis tissues. Positive (negative) logFC indicates that the gene was up-regulated (down-regulated), where gene expression was greater (reduced) in the stressed group than control group.

| Tissue | Ensembl ID | Gene | logFC | FDR |
|---|---|---|---|---|
| Paraventricular nucleus | ENSBTAG00000005353 | *Des* | 3.900 | 0.050 |
| Paraventricular nucleus | ENSBTAG00000015988 | *Myh11* | 3.235 | 0.051 |
| Paraventricular nucleus | ENSBTAG00000014614 | *Acta2* | 2.659 | 0.061 |
| Paraventricular nucleus | ENSBTAG00000032531 | *Mustn1* | 3.111 | 0.061 |
| Paraventricular nucleus | ENSBTAG00000011473 | *Myl9* | 2.251 | 0.141 |
| Paraventricular nucleus | ENSBTAG00000007196 | *Tagln* | 2.438 | 0.141 |
| Anterior pituitary | ENSBTAG00000013400 | *Vip* | 2.325 | 0.008 |
| Anterior pituitary | ENSBTAG00000009190 | *Slc2a4* | 1.456 | 0.008 |
| Anterior pituitary | ENSBTAG00000017504 | *Faim2* | -0.896 | 0.028 |
| Anterior pituitary | ENSBTAG00000018576 | *Dpysl5* | -0.846 | 0.028 |
| Anterior pituitary | ENSBTAG00000003635 | *Slc35c2* | 0.626 | 0.028 |
| Anterior pituitary | ENSBTAG00000005857 | *Slc6a1* | -1.360 | 0.028 |
| Anterior pituitary | ENSBTAG00000044092 | *Iqck* | -0.990 | 0.028 |
| Anterior pituitary | ENSBTAG00000019822 | *Tppp3* | -0.740 | 0.036 |
| Anterior pituitary | ENSBTAG00000015715 | *Spocd1* | 2.445 | 0.039 |
| Anterior pituitary | ENSBTAG00000045887 | *Dmtn* | -0.679 | 0.076 |
| Anterior pituitary | ENSBTAG00000012889 | *Neto1* | 1.865 | 0.076 |
| Anterior pituitary | ENSBTAG00000006977 | *Plp1* | -2.904 | 0.076 |
| Anterior pituitary | ENSBTAG00000001517 | *Krt18* | 1.061 | 0.076 |
| Anterior pituitary | ENSBTAG00000014312 | *Atl1* | -0.694 | 0.076 |
| Anterior pituitary | ENSBTAG00000051072 | *Aqp4* | -1.431 | 0.079 |
| Anterior pituitary | ENSBTAG00000043553 | *Gpx3* | 0.634 | 0.079 |
| Anterior pituitary | ENSBTAG00000018653 | *Nabp1* | 0.834 | 0.079 |
| Anterior pituitary | ENSBTAG00000001703 | *Syt13* | -0.623 | 0.080 |
| Anterior pituitary | ENSBTAG00000008739 | *Samd11* | -0.752 | 0.080 |
| Anterior pituitary | ENSBTAG00000012302 | *Rtn4rl1* | -1.034 | 0.080 |

| | | | | |
|---|---|---|---|---|
| Anterior pituitary | ENSBTAG00000013792 | *Ubald2* | 0.905 | 0.080 |
| Anterior pituitary | ENSBTAG00000054523 | *Cnr1* | -0.653 | 0.080 |
| Anterior pituitary | ENSBTAG00000043490 | *Novel* | 0.731 | 0.080 |
| Anterior pituitary | ENSBTAG00000055216 | *Novel* | -1.564 | 0.080 |
| Anterior pituitary | ENSBTAG00000019011 | *Pgm1* | 0.684 | 0.080 |
| Anterior pituitary | ENSBTAG00000013534 | *Gfap* | -2.730 | 0.086 |
| Anterior pituitary | ENSBTAG00000005304 | *Srm* | 0.705 | 0.103 |
| Anterior pituitary | ENSBTAG00000047650 | *Nxph4* | -0.565 | 0.103 |
| Anterior pituitary | ENSBTAG00000000836 | *Krt8* | 0.677 | 0.103 |
| Anterior pituitary | ENSBTAG00000013054 | *Mfsd2a* | 0.783 | 0.103 |
| Anterior pituitary | ENSBTAG00000019399 | *Cnksr1* | 1.232 | 0.103 |
| Anterior pituitary | ENSBTAG00000010152 | *Mid1* | 0.565 | 0.103 |
| Anterior pituitary | ENSBTAG00000016045 | *Trib2* | 0.664 | 0.103 |
| Anterior pituitary | ENSBTAG00000045547 | *Amigo3* | 0.701 | 0.121 |
| Anterior pituitary | ENSBTAG00000002201 | *Nfxl1* | 0.616 | 0.121 |
| Anterior pituitary | ENSBTAG00000000920 | *Dnah17* | 0.870 | 0.121 |
| Anterior pituitary | ENSBTAG00000022890 | *Mbp* | -3.044 | 0.121 |
| Anterior pituitary | ENSBTAG00000050334 | *Cd83* | -0.897 | 0.121 |
| Anterior pituitary | ENSBTAG00000001060 | *Cxcr4* | -0.509 | 0.121 |
| Anterior pituitary | ENSBTAG00000019267 | *Mmp2* | -0.649 | 0.121 |
| Anterior pituitary | ENSBTAG00000009302 | *Rcan2* | -0.627 | 0.125 |
| Anterior pituitary | ENSBTAG00000000564 | *Nsg2* | -2.220 | 0.125 |
| Anterior pituitary | ENSBTAG00000013662 | *Col8a1* | 0.916 | 0.128 |
| Anterior pituitary | ENSBTAG00000015963 | *Dmac1* | 0.515 | 0.130 |
| Anterior pituitary | ENSBTAG00000019031 | *Plppr4* | -0.975 | 0.131 |
| Anterior pituitary | ENSBTAG00000017375 | *Fam241a* | 0.820 | 0.136 |
| Anterior pituitary | ENSBTAG00000051461 | *Sertm1* | -0.603 | 0.137 |
| Anterior pituitary | ENSBTAG00000004515 | *Kcnk1* | 0.571 | 0.138 |
| Anterior pituitary | ENSBTAG00000021818 | *Adgre5* | 0.641 | 0.144 |

| | | | | |
|---|---|---|---|---|
| Adrenal cortex | ENSBTAG00000021957 | *Ltbp2* | 0.895 | 0.003 |
| Adrenal cortex | ENSBTAG00000015274 | *Prl* | 1.728 | 0.003 |
| Adrenal cortex | ENSBTAG00000017220 | *Gh1* | 1.565 | 0.008 |
| Adrenal cortex | ENSBTAG00000026497 | *A4galt* | 0.657 | 0.043 |
| Adrenal cortex | ENSBTAG00000017280 | *C3* | 0.925 | 0.074 |

**Supplementary Table S3.** Gene ontology and KEGG pathway functional annotation results for differentially expressed genes within the paraventricular nucleus of the stress axis tissues.

| **Paraventricular nucleus** | **Functional Category** | **Term** | **Genes Involved** |
|---|---|---|---|
| Differentially expressed genes | GO: Cellular Component | Stress fiber | *Acta2, Myl9* |
| | GO: Cellular Component | Z disc | *Des, Myl9* |
| | GO: Molecular Function | Actin filament binding | *Myh11, Tagln* |
| | GO: Biological Process | Positive regulation of gene expression | *Acta2, Mustn1* |
| | KEGG Pathway | Tight junction | *Myh11, Myl9* |
| | KEGG Pathway | Regulation of actin cytoskeleton | *Myh11, Myl9* |
| | KEGG Pathway | Vascular smooth muscle contraction | *Acta2, Myh11, Myl9* |

**Supplementary Table S4.** Gene ontology and KEGG pathway functional annotation results for differentially methylated genes and differentially expressed genes within the anterior pituitary of the stress axis tissues.

| Anterior pituitary | Functional Category | Term | Genes Involved |
|---|---|---|---|
| Differentially methylated genes | GO: Cellular Component | Cytoplasm | *Brinp1, Cdc42bpb, Crtc1,Gnb1, Nedd4l, Traf3, Capzb, Cdyl, Hipk2, Ighmbp2, Kif5c, Kat2b, Msra, Mcm3ap, Mad1l1, Prkn, Phactr1, Pfkp, Smap1, Tecpr2, Ubash3b* |
| | GO: Cellular Component | Ruffle membrane | *Rac1, Egfr, Hip1r* |
| | GO: Cellular Component | Actomyosin | *Cdc42bpb, Kat2b* |
| | GO: Cellular Component | WASH complex | *Washc5, Capzb* |
| | GO: Cellular Component | Cytosol | *Baiap2l1, Crtc1, Eml1, Rac1, Anks1b, Cryl1, Flna, Hip1r, Kat2b, Msra, Mcm3ap, Prkn, Phactr1, Pald1, Ppm1f, Rara* |
| | GO: Cellular Component | Nuclear pore nuclear basket | *Mcm3ap, Mad1l1* |
| | GO: Cellular Component | Axonal growth cone | *Flna, Kif5c* |
| | GO: Cellular Component | Perinuclear region of cytoplasm | *Abcd1, Adgrb1, Egfr, Hip1r, Prkn* |
| | GO: Cellular Component | Nucleus | *Bcl11b, Crtc1, Rac1, Cdyl, Egfr, Hipk2, Ighmbp2, Irf2bpl, Lpin3, Kat2b, Mcm3ap, Mad1l1, Phactr1, Ppm1f, Rara, Rarb, Tshz2, Znf516* |
| | GO: Cellular Component | Lamellipodium | *Cdc42bpb, Rac1, Capzb* |

| | | |
|---|---|---|
| GO: Cellular Component | Cytoskeleton | *Cdc42bpb, Eml1, Rac1, Hip1r* |
| GO: Cellular Component | Mitotic spindle pole | *Eml1, Mad1l1* |
| GO: Cellular Component | Actin cytoskeleton | *Baiap2l1, Msra, Rara* |
| GO: Molecular Function | Macromolecular complex binding | *Cdc42bpb, Gnb1, Rac1, Prkn, Pfkp* |
| GO: Molecular Function | Transcription corepressor activity | *Cdyl, Hipk2, Irf2bpl, Prkn* |
| GO: Molecular Function | GTPase activity | *Gnb1, Rac1* |
| GO: Molecular Function | Actin filament binding | *Capzb, Egfr, Flna, Hip1r* |
| GO: Molecular Function | Thioesterase binding | *Rac1, Traf3* |
| GO: Molecular Function | Actin binding | *Capzb, Lsp1, Prkn, Phactr1* |
| GO: Molecular Function | Protein tyrosine phosphatase activity | *Dusp13, Pald1, Ubash3b* |
| GO: Molecular Function | Protein tyrosine/serine/threonine phosphatase activity | *Dusp13, Ppm1f* |
| GO: Molecular Function | Helicase activity | *Ighmbp2, Zranb3* |
| GO: Molecular Function | Ubiquitin-protein transferase activity | *Nedd4l, Traf3, Prkn* |
| GO: Biological Process | Actin cytoskeleton organization | *Cdc42bpb, Rac1, Capzb, Flna, Phactr1* |
| GO: Biological Process | Positive regulation of transcription from RNA polymerase II promoter | *Bcl11b, Crtc1, Egfr, Hipk2, Irf2bpl, Lpin3, Kat2b, Prkn, Rara, Rarb* |

| | | |
|---|---|---|
| GO: Biological Process | Negative regulation of protein catabolic process | *Adgrb1, Egfr, Flna* |
| GO: Biological Process | Glandular epithelial cell development | *Rara, Rarb* |
| GO: Biological Process | Ventricular cardiac muscle cell differentiation | *Rara, Rarb* |
| GO: Biological Process | Regulation of cellular response to oxidative stress | *Abcd1, Prkn* |
| GO: Biological Process | Regulation of clathrin-dependent endocytosis | *Hip1r, Smap1* |
| GO: Biological Process | Growth plate cartilage development | *Rara, Rarb* |
| GO: Biological Process | Positive regulation of protein binding | *Hipk2, Hip1r, Prkn* |
| GO: Biological Process | Vocalization behavior | *Brinp1, Nrxn2* |
| GO: Biological Process | Actin crosslink formation | *Baiap2l1, Flna* |
| GO: Biological Process | Retinoic acid receptor signaling pathway | *Rara, Rarb* |
| GO: Biological Process | Positive regulation of dendrite Extension | *Nedd4l, Prkn* |
| GO: Biological Process | Negative regulation of chondrocyte differentiation | *Adamts7, Rarb* |
| GO: Biological Process | Positive regulation of DNA binding | *Hipk2, Prkn* |
| GO: Biological Process | Engulfment of apoptotic cell | *Rac1, Adgrb1* |
| GO: Biological Process | Positive regulation of focal adhesion assembly | *Rac1, Ppm1f* |
| GO: Biological Process | Motor neuron axon guidance | *Rac1, Kif5c* |

| | | | |
|---|---|---|---|
| | GO: Biological Process | Cell motility | *Rac1, Phactr1* |
| | GO: Biological Process | Semaphorin-plexin signaling pathway | *Rac1, Flna* |
| | GO: Biological Process | Lamellipodium assembly | *Rac1, Capzb* |
| | GO: Biological Process | Heart morphogenesis | *Col5a1, Flna* |
| | GO: Biological Process | Ureteric bud development | *Rara, Rarb* |
| | KEGG Pathway | Endocytosis | *Nedd4l, Washc5, Capzb, Egfr, Kif5c, Smap1* |
| | KEGG Pathway | Non-small cell lung cancer | *Egfr, Kif5c, Rarb* |
| | KEGG Pathway | Kaposi sarcoma-associated herpesvirus infection | *Gnb1, Rac1, Traf3, Pik3r6* |
| | KEGG Pathway | Pathways in cancer | *Gnb1, Rac1, Traf3, Egfr, Rara, Rarb* |
| | KEGG Pathway | Viral carcinogenesis | *Rac1, Traf3, Kat2b, Mad1l1* |
| Differentially expressed genes | GO: Cellular Component | Integral component of membrane | *Adgre5, Aqp4, Mfsd2a, Plppt4, Kcnk1. Slc2a4, Slc6a1, Syt13* |
| | GO: Cellular Component | Astrocyte end-foot | *Aqp4, Gfap* |
| | GO: Cellular Component | Sarcolemma | *Aqp4, Krt8, Slc2a4* |
| | GO: Cellular Component | Neuronal cell body | *Dpysl5, Mbp, Slc6a1* |
| | GO: Cellular Component | Myelin sheath | *Mbp, Plp1* |
| | GO: Cellular Component | Anchoring junction | *Cxcr4, Faim2, Kcnk1* |

| | | |
|---|---|---|
| GO: Cellular Component | Cell periphery | *Krt18, Mbp* |
| GO: Cellular Component | Postsynaptic density membrane | *Neto1, Plppr4* |
| GO: Cellular Component | Axon | *Atl1, Slc6a1, Syt13* |
| GO: Cellular Component | Glutamatergic synapse | *Cnr1, Neto1, Plppr4* |
| GO: Cellular Component | Extracellular region | *Aqp4, Col8a1, Mmp2, Nxph4, Vip* |
| GO: Cellular Component | Dendrite | *Dpysl5, Nsg2, Kcnk1* |
| GO: Molecular Function | Structural constituent of myelin sheath | *Mbp, Plp1* |
| GO: Molecular Function | Ubiquitin protein ligase binding | *Cxcr4, Mid1, Trib2* |
| GO: Molecular Function | Scaffold protein binding | *Krt18, Krt8* |
| GO: Biological Process | Maintenance of permeability of blood-brain barrier | *Mfsd2a, Mbp* |
| GO: Biological Process | Hepatocyte apoptotic process | *Krt18, Krt8* |
| GO: Biological Process | Astrocyte development | *Gfap, Plp1* |
| GO: Biological Process | Extracellular matrix organization | *Col8a1, Gfap, Mmp2* |
| GO: Biological Process | Endodermal cell differentiation | *Col8a1, Mmp2* |
| GO: Biological Process | Embryo implantation | *Mmp2, Tppp3* |
| GO: Biological Process | Extrinsic apoptotic signaling pathway | *Krt18, Krt8* |

| | | | |
|---|---|---|---|
| | GO: Biological Process | Tumor necrosis factor-mediated signaling pathway | *Krt18, Krt8* |
| | GO: Biological Process | Myelination | *Mbp, Plp1* |

**Supplementary Table S5.** Gene ontology and KEGG pathway functional annotation results for differentially methylated genes and differentially expressed genes within the adrenal cortex of the stress axis tissues.

| Adrenal cortex | Functional Category | Term | Genes Involved |
|---|---|---|---|
| Differentially methylated genes | GO: Cellular Component | Presynaptic membrane | *Grin2a, Grik4* |
| | GO: Cellular Component | NMDA selective glutamate receptor complex | *Grin2a* |
| | GO: Cellular Component | Synaptic vesicle | *Grin2a, Kirrel3* |
| | GO: Cellular Component | Glutamatergic synapse | *Ctbp1, Grin2a, Prkar1b* |
| | GO: Cellular Component | Protein phosphatase type 2A complex | *Ppp2r3b, Ppp2r2c* |
| | GO: Cellular Component | Postsynaptic membrane | *Grin2a, Grik4* |
| | GO: Cellular Component | Neuron projection | *Grin2a, Kcnq1, Ptprf* |
| | GO: Cellular Component | Myelin sheath | *Bcl2, Mbp* |
| | GO: Cellular Component | Apical junction complex | *Fbf1, Pard3* |
| | GO: Cellular Component | Adherens junction | *Ctnnd2, Pard3, Tmem204* |
| | GO: Cellular Component | Hippocampal mossy fiber to CA3 synapse | *Grik4, Prkar1b* |
| | GO: Molecular Function | Ligand-gated ion channel activity | *Grin2a, Grik4* |
| | GO: Molecular Function | Glutamate-gated calcium ion channel activity | *Grin2a* |

| | | |
|---|---|---|
| GO: Molecular Function | Heparin binding | *Hbegf, Nav2, Ptprf, Tnxb* |
| GO: Molecular Function | NMDA glutamate receptor activity | *Grin2a* |
| GO: Molecular Function | cAMP-dependent protein kinase inhibitor activity | *Prkag2, Prkar1b* |
| GO: Molecular Function | Protein kinase A catalytic subunit binding | *Kcnq1, Prkar1b* |
| GO: Molecular Function | Ionotropic glutamate receptor activity | *Grin2a* |
| GO: Molecular Function | Serine-type peptidase activity | *Dpp9, Dpp6* |
| GO: Molecular Function | Signaling receptor activity | *Grin2a, Grik4* |
| GO: Molecular Function | Protein phosphatase 1 binding | *Kcnq1, Ppp1r3g* |
| GO: Biological Process | Cell-cell adhesion | *Bcl2, Celsr1, Ctnnd2, Kirrel3, Tnxb* |
| GO: Biological Process | Synapse assembly | *Kirrel3, Plxnd1, Sdk1* |
| GO: Biological Process | Long-term synaptic potentiation | *Crhr2, Grin2a* |
| GO: Biological Process | Neuron migration | *Satb2, Celsr1, Dcdc2, Kirrel3* |
| GO: Biological Process | Neurogenesis | *Grin2a, Nav2* |
| GO: Biological Process | Protein localization to postsynaptic membrane | *Grin2a* |
| GO: Biological Process | Directional locomotion | *Grin2a* |
| GO: Biological Process | Sleep | *Grin2a* |

| | | |
|---|---|---|
| GO: Biological Process | Serotonin metabolic process | *Grin2a* |
| GO: Biological Process | Calcium ion transmembrane import into cytosol | *Grin2a* |
| GO: Biological Process | Dopamine metabolic process | *Grin2a* |
| GO: Biological Process | Response to drug | *Bcl2, Grin2a* |
| GO: Biological Process | Response to nicotine | *Bcl2, Kcnq1* |
| GO: Biological Process | Startle response | *Grin2a* |
| GO: Biological Process | Response to ethanol | *Grin2a* |
| GO: Biological Process | Cellular response to organic substance | *Bcl2, Satb2* |
| GO: Biological Process | Regulation of neurotransmitter secretion | *Cplx1, Mctp1* |
| GO: Biological Process | Exocytosis | *Cplx1, Rph3al, Sytl3* |
| GO: Biological Process | Response to amphetamine | *Grin2a* |
| GO: Biological Process | Protein dephosphorylation | *Bcl2, Ppp2r3c, Ptprf* |
| GO: Biological Process | Osteoblast development | *Lrp5, Satb2* |
| GO: Biological Process | Regulation of cilium assembly | *Dcdc2, Ift140* |
| KEGG Pathway | Sphingolipid signaling pathway | *Bcl2, Degs2, Ppp2r3b, Ppp2r2c* |
| KEGG Pathway | Adrenergic signaling in cardiomyocytes | *Bcl2, Kcnq1, Ppp2r3b, Ppp2r2c* |

| | | | |
|---|---|---|---|
| | KEGG Pathway | Wnt signaling pathway | *Ctbp1, Lrp5, Ctnnd2, Lgr6* |
| | KEGG Pathway | Fc gamma R-mediated phagocytosis | *Fcgr2b, Dock1, Inpp5d* |
| | KEGG Pathway | Parathyroid hormone synthesis, secretion and action | *Bcl2, Lrp5, Hbegf* |
| | KEGG Pathway | PI3K-Akt signaling pathway | *Bcl2, Angpt2, Ppp2r3b, Ppp2r2c, Tnxb* |
| | KEGG Pathway | Glutamatergic synapse | *Shank2, Grin2a, Grik4* |
| Differentially expressed genes | GO: Cellular Component | Extracellular region | *C3, Ltbp2, Prl* |
| | GO: Cellular Component | Extracellular space | *C3, Gh1, Prl* |
| | GO: Molecular Function | Hormone activity | *Gh1, Prl* |
| | GO: Biological Process | Response to L-arginine | *Gh1, Prl* |
| | GO: Biological Process | Positive regulation of fatty acid biosynthetic process | *Gh1, Prl* |
| | GO: Biological Process | Positive regulation of lactation | *Gh1, Prl* |
| | GO: Biological Process | Response to nutrient levels | *Gh1, Prl* |
| | GO: Biological Process | Response to food | *Gh1, Prl* |
| | GO: Biological Process | Positive regulation of JAK-STAT cascade | *Gh1, Prl* |
| | GO: Biological Process | Negative regulation of gene expression | *Gh1, Prl* |
| | GO: Biological Process | Negative regulation of apoptotic process | *Gh1, Prl* |

| | | |
|---|---|---|
| GO: Biological Process | Positive regulation of gene expression | *Gh1, Prl* |
| KEGG Pathway | Neuroactive ligand-receptor interaction | *C3, Gh1, Prl* |
| KEGG Pathway | JAK-STAT signaling pathway | *Gh1, Prl* |
